# Structural Elucidation of Novel Saponins in the Sea Cucumber *Holothuria lessoni*

**DOI:** 10.3390/md12084439

**Published:** 2014-08-08

**Authors:** Yadollah Bahrami, Wei Zhang, Tim Chataway, Chris Franco

**Affiliations:** 1 Department of Medical Biotechnology, School of Medicine, Flinders University, Adelaide, SA 5042, Australia; E-Mails: yadollah.bahrami@flinders.edu.au (Y.B.); wei.zhang@flinders.edu.au (W.Z.); 2 Centre for Marine Bioproducts Development, Flinders University, Adelaide, SA 5042, Australia; 3 Australian Seafood Cooperative Research Centre, Mark Oliphant Building, Science Park, Adelaide SA 5042, Australia; 4 Medical Biology Research Center, Kermanshah University of Medical Sciences, Kermanshah 6714415185, Iran; 5 Flinders Proteomics Facility, School of Medicine, Flinders University, Adelaide, SA 5042, Australia; E-Mail: tim.chataway@flinders.edu.au

**Keywords:** sea cucumber viscera, saponins, bioactive compounds, MALDI, ESI, mass spectrometry, HPCPC, triterpene glycosides, structure elucidation, marine invertebrate, Echinodermata, holothurian

## Abstract

Sea cucumbers are prolific producers of a wide range of bioactive compounds. This study aimed to purify and characterize one class of compound, the saponins, from the viscera of the Australian sea cucumber *Holothuria lessoni*. The saponins were obtained by ethanolic extraction of the viscera and enriched by a liquid-liquid partition process and adsorption column chromatography. A high performance centrifugal partition chromatography (HPCPC) was applied to the saponin-enriched mixture to obtain saponins with high purity. The resultant purified saponins were profiled using MALDI-MS/MS and ESI-MS/MS which revealed the structure of isomeric saponins to contain multiple aglycones and/or sugar residues. We have elucidated the structure of five novel saponins, Holothurins D/E and Holothurinosides X/Y/Z, along with seven reported triterpene glycosides, including sulfated and non-sulfated saponins containing a range of aglycones and sugar moieties, from the viscera of *H. lessoni*. The abundance of novel compounds from this species holds promise for biotechnological applications.

## 1. Introduction

Holothurians vary in size, shape and color, and belong to the class Holothuroidea of the *Echinodermata* phylum [1]. Sea cucumbers are known to produce a range of compounds which are present in agricultural or agrochemical, nutraceutical, pharmaceutical and cosmeceutical products [2,3,4,5].

Sea cucumbers are used in traditional Asian medicine to treat diseases such as rheumatoid arthritis, joint-pain, cardiovascular, tendonitis, gastric, osteoarthritis, ankylosing spondylitis, arthralgia, tumors, fungal infection, impotence, frequent urination and kidney deficiency, high blood pressure, arthritis and muscular disorders [6,7,8,9,10] and are also used as a general tonic [9,11,12]. Of the many chemical classes present in sea cucumbers, saponins are the most important and abundant secondary metabolites [13,14,15,16,17,18,19]. They are generally perceived as highly active natural products and the sea cucumber saponins have been well characterized for their biological activities. They possess a wide range of therapeutic applications due to their cardiovascular, immunomodulator, cytotoxic, anti-asthma, anti-eczema, anti-inflammatory, anti-arthritis, anti-oxidant, anti-diabetics, anti-bacterial, anti-viral, anti-cancer, anti-angiogenesis, anti-fungal, hemolytic, cytostatic, cholesterol-lowering, hypoglycemia and anti-dementia activities [3,12,14,20,21,22,23,24,25,26,27,28,29,30,31,32,33].

Saponins are produced by a limited number of marine species which belong to the phylum *Echinodermata* [34], namely holothuroids (sea cucumbers) [14,17,20,35,36,37,38,39,40,41], asteroids, and sponges from the phylum *Porifera* [20,42,43]. They are amphipathic compounds that generally possess a triterpene or steroid backbone or aglycone which in sea cucumbers is of the holostane type [44,45]. Although sea cucumber saponins usually share common features, their aglycones, also called sapogenins or genins, are significantly different from those reported in the plant kingdom [3]. They comprise a lanostane-3β-ol type aglycone containing a γ-18 (20)-lactone in the d-ring of tetracyclic triterpene (3β,20*S*-dihydroxy-5α-lanostano-18,20-lactone) [46] and can contain shortened side chains and a carbohydrate moiety consisting of up to six monosaccharide units covalently connected to C-3 of the aglycone [14,15,20,45,47,48,49,50,51].

The sugar moiety of the sea cucumber saponins consists principally of d-xylose, d-quinovose, 3-*O-*methyl-d-glucose, 3-*O*-methyl-d-xylose and d-glucose and less frequently 3-*O*-methyl-d-quinovose, 3-*O*-methyl-d-glucuronic acid and 6-*O*-acetyl-d-glucose [47,49,52,53,54,55,56,57]. In the oligosaccharide chain, the first monosaccharide unit is always a xylose, whereas 3-*O-*methylglucose or 3-*O*-methylxylose are always the terminal sugars. A plant saponin may contain one, two or three saccharide chains, often with an acyl group bound to the sugar moiety [58], whereas, in sea cucumbers, the sugar residue has only one branch [49].

Over 500 triterpene glycosides have been reported from various species of sea cucumbers [11,12,14,20,25,36,46,47,55,59] and are classified into four main structural categories based on their aglycone moieties; three holostane type glycoside group saponins containing a (1) 3β-hydroxyholost-9 (11)-ene aglycone skeleton; (2) saponins with a 3β-hydroxyholost-7-ene skeleton; and (3) saponins with an aglycone moiety different to the other two holostane type aglycones (other holostane type aglycones); and (4) a nonholostane aglycone [46,48,54,60,61].

Many of the saponins from marine organisms have sulfated aglycones or sugar moieties [3]. Sulfation of the oligosaccharide chain in the Xyl, Glc and MeGlc residues have been reported in sea cucumber saponins [48,49,54,62,63]. Most of them are mono-sulfated glycosides with few occurrences of di- and tri-sulfated glycosides. Saponin diversity can be further enhanced by the position of double bonds and lateral groups in the aglycone.

The most commonly accepted biological role for these secondary metabolites in nature is that they are a powerful defense mechanism for sea cucumbers as they are deleterious for most organisms [13,14,15,16,17,18,64,65,66] and are responsible for the organism’s environmental defense mechanisms in general. Sea cucumbers expel their internal organs as a defense mechanism called evisceration, a reaction that includes release of the respiratory tree, intestine, cuvierian tubules and gonads through the anal opening [59,67,68,69,70,71,72,73,74]. The deterrent effect of saponins seems, therefore, to act as an aposematic signal, warning potential predators of the unpalatability of the holothuroid tissues [70]. In contrast, a recent study has shown that these repellent chemicals are also kairomones attracting the symbionts and are used as chemical “communicates” [67]. However, in the sea cucumber, it was suggested that saponins may also have two regulatory roles during reproduction: (1) to prevent oocyte maturation and (2) to act as a mediator of gametogenesis [25,75].

In this paper we describe the structural characterization of novel bioactive triterpene glycosides from the viscera (which comprises all internal organs other than the body wall) of an Australian sea cucumber *Holothuria lessoni* (golden sandfish) [76]. *H. lessoni* is a newly-identified Holothurian species, which is abundant in Australian waters. We hypothesize that the reason for their ingenious form of defense is because their internal organs contain high levels of compounds that repel predators [72,77,78,79]. The results of this project will assist in transforming viscera of the sea cucumber into high value co-products, important to human health and industry.

We have used matrix-assisted laser desorption/ionization mass spectrometry (MALDI-MS) and electrospray ionization mass spectrometry (ESI-MS), and MS/MS to elucidate the structure of five novel isomeric saponins. Knowledge of the chemical structure of compounds is very important for determining the specific correlation between the structure and their molecular and biological mechanism(s) of actions [22,25,28,46].

## 2. Results and Discussion

Several saponins were isolated and purified from the viscera of *H. lessoni* sea cucumber species using HPCPC. The extraction and purification procedures and the mass spectrometry analysis was described in detail previously [12]. Mass spectrometry has been applied for the structure elucidation of saponins in both negative and positive ion modes [80,81,82,83,84,85,86]. In this study, identification of the sugar component of saponin compounds was performed by soft ionization MS techniques including MALDI and ESI in positive ion mode. The low CID energy MS/MS techniques used here do not fragment the aglycone. In addition, LC was not used to separate the compounds before introduction into the mass spectrometer. Instead high performance centrifugal partition chromatography (HPCPC) was conducted which we believe is more efficient for the separation of saponins. Identification of the aglycone component of the saponins was performed by comparison with published data. In these papers, the structure elucidation of the aglycones was confirmed predominantly by NMR which is capable of determining detailed structural analysis. Therefore, while we are confident of the assignment of the aglycones, confirmation of these assignments should be made by NMR. We have previously highlighted the presence of isomers in the saponin mixture [12]. The MS analysis was conducted by introducing sodium ions to the samples. Because of the high affinity of alkali cations for triterpene glycosides, all saponins detected in the positive ion mode spectra were predominantly singly charged sodium adducts of the molecules [M + Na]^+^. The main fragmentation of saponins generated by cleavage of the glycosidic bond yielded oligosaccharide and monosaccharide fragments [24]. Other visible peaks and fragments were generated by the loss of other neutral moieties such as CO_2_, H_2_O or CO_2_ coupled with H_2_O.

The appropriate HPCPC fractions were pooled based on their thin-layer chromatography (TLC) profiles (Supplementary Figure S1), concentrated to dryness, and analyzed by MALD-MS/MS and ESI-MS/MS. The ESI and MALDI spectra reflect the saponin profile of each HPCPC fraction.

### 2.1. Structure Elucidation of Saponins by ESI-MS

**Figure 1:**
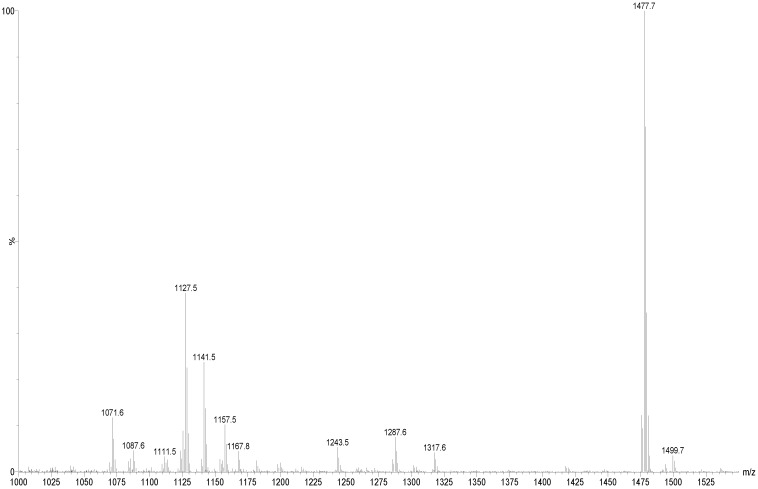
(+) Electrospray ionization- mass spectrometry (ESI-MS) spectrum of saponins purified by HPCPC from Fraction # 18 of the extract from the viscera of *H. lessoni*.

ESI-MS^n^ is a very effective and powerful technique to differentiate isomeric saponins as they exhibit different MS^n^ fingerprints spectra [77,87,88]. ESI-MS/MS analysis was conducted on all saponin ions detected in the ESI-MS spectrum of the HPCPC fractions in positive ion mode. ESI mass spectra of the saponins are dominated by [M + Na]^+^. The ESI-MS spectrum of the saponin extract from Fraction 18 of the viscera of *H. lessoni* is shown in Figure 1.

Fifteen major peaks were detected which correspond to several novel and known triterpene compounds. It is notable that the observed ions generate from cationization of the neutral molecules in MALDI or ESI. The ions at *m/z* 1071.6 (Unidentified), 1087.6 (Unidentified), 1125.5 (Holothurinosides C/C_1)_, 1141.5 (Desholothurin A_1_ and Desholothurin A (synonymous with Nobiliside 2A), 1157.5 (Holothurinoside J_1_), 1227.4 (Fuscocinerosides B/C or Scabraside A or 24-dehydroechinoside A and a novel saponin), 1243.5 (Holothurin A), 1287.6 (Holothurinosides E/E_1_/O/P), 1301.6 (Holothurinosides M), 1303.6 (Holothurinosides A/A_1_/Q/R/R_1_/S), 1305.4 (Unidentified), 1317.6 (Holothurinoside N), 1417.7 (Unidentified), 1477.7 (Unidentified), 1479.7 (Holothurinoside I), 1493.7 (Unidentified) and 1495.7 (Holothurinoside K_1_) were detected. The spectrum displays one dominant peak at *m/z* 1477.7 which corresponds to an unidentified (novel) saponin(s), with an elemental composition of C_61_H_114_O_38_ which requires further analysis and will be published later. The names, elemental compositions and producing organisms of saponins detected in this fraction are summarized in Table 1. Further analysis revealed that some of these peaks represented more than one compound. The ions at *m/z* 1227.4, 1229.5, 1243.5, and 1259.5 are sulfated compounds, whereas the ion peaks at *m/z* 1125.5, 1141.5, 1287.6, 1301.6 and 1303.6 correspond to the non-sulfated saponins.

Thus Fraction 18 contains several saponin congeners indicating that complete separation of the saponins was not possible within a single HPCPC run due to the high similarity of their structures. However, this technique allowed the separation of a number of saponins, including some isomers.

Our method to elucidate or propose the molecular structure of saponins was based on the MS/MS spectra as described in detail in Bahrami *et al*. [12]. Firstly, fragmentation patterns were built, and the generated fingerprint signals were reconstructed according to the measurement of the mass transitions between the successive collision-activated fragmentation signals. In other words, based on MS/MS spectra the molecular structures of the saponins were obtained by the identification of the mass transitions between the successive collision-induced fragmentation peaks.

The ESI and MALDI mass spectrum of the isobutanol-enriched saponin extract obtained from the viscera of the *H. lessoni* shows a diverse range of saponins [12]. Our results revealed that at least 75 saponins (29 sulfated and 46 non-sulfated) were detected in *H. lessoni*, including 39 new sulfated, non-sulfated and acetylated triterpene glycosides, containing a wide range of aglycone and sugar moieties of which 36 congeners were previously identified in other holothurians.

In this manuscript we describe the structure elucidation of ions at *m/z* 1127.6, 1227.4, 1243.5 and 1259.5 (from HPCPC Fractions 17, 18, 20 and 22). Among these saponins, Holothurin A is the reported major congener with the highest relative abundance in this species.

Table 1 summarizes the data of all analysis performed on the HPCPC fraction 18 using MALDI-MS and ESI-MS on compounds. This fractionated sample contained 14 novel saponins along with 27 reported triterpene glycosides, including 14 sulfated and 27 non-sulfated saponin ions.

**Table 1:** Summary of saponins identified from Fraction 18 of the viscera of *H. lessoni* by MALDI-MS/MS and ESI-MS/MS. This table includes the 14 novel identified compounds (N) along with the 27 published compounds (P).

[M + Na]^+^ *m/z*	MW	Formula	Compound’s Name	Novel (N)/ Published(P)	Sea Cucumber Species A. = *Actinopyga*; B. = *Bohadschia*; P. = *Pearsonothuria*; H. = *Holothuria*	References
1071.6	1048	C_47_H_93_NaO_21_S	Unidentified	N	*H. lessoni*	[12]
1083.3	1060	C_58_H_64_O_25_	Unidentified	N	*H. lessoni*	[12]
1087.6	1064	C_47_H_93_NaO_22_S	Unidentified	N	*H. lessoni*	[12]
1123.5	1100	C_54_H_84_O_23_	Unidentified	N	*H. lessoni*	[12]
1125.5	1102	C_54_H_86_O_23_	Holothurinoside C	P	*H. lessoni*, *H. forskali*, *A. agassizi*, *H. scabra*, *H. fuscocinerea* and *H. impatiens*	[12,67,69,77,89,90]
			Holothurinoside C_1_	P		
1127.6	1104	C_54_H_88_O_23_	Unidentified	N	*H. lessoni*	-
			Unidentified	N	*H. lessoni*	-
			Unidentified	N	*H. lessoni*	-
1141.5	1118	C_54_H_86_O_24_	Desholothurin A	P	*H. lessoni*, *H. forskali*, *H. nobilis*, *A. agassizi*, *B. argus*, *B. cousteaui*, *H. leucospilota*, *P. graeffei*	[4,12,13,77,89,90,91,92,93]
			(Nobiliside 2a),	P		
			Desholothurin A_1_ (Arguside E)	P		
1149.2	1126	a *	Holothurinoside T	P	*H. lessoni*	[12]
1157.5	1134	C_54_H_109_O_25_	Holothurinoside J_1_	P	*H. lessoni*, *B. subrubra*	[12,59]
		C_49_H_91_NaO_25_S	Unidentified	N	*H. lessoni*	-
1169.5	1170	C_55_H_87_NaO_23_S	Unidentified	N	*H. lessoni*	-
1227.4	1204	C_54_H_85_NaO_26_S	Fuscocinerosides B/C, Scabraside A or 24–dehydroechinoside A, Unidentified	P	*B. subrubra*, *H. lessoni*, *H. scabra*, *H. leucospilota*, *H. fuscocinerea*, *A. agassizi*, and *H. impatiens*, *P. graeffei*, *A. echinites*	[12,13,36,64,67,69,90,94,95,96]
				P		
				N		
1243.5	1220	C_54_H_85_NaO_27_S	Holothurin A Scabraside B 17-Hydroxy fuscocineroside B 25-Hydroxy fuscocinerosiden B	P P P P	*H. lessoni*, *H. scabra*, *H. atra*, *H. leucospilota*, *H. arenicola*, *H. cinerascens*, *H. coluber*, *H. cubana, H. difficilis*, *H. gracilis*, *H. pervicax*, *H. lubrica*, *H. polii*, *H. pulla*, *H. squamifera*, *H. surinamensis*, *H. tubulosa*, *P. graeffei*, *A. agassizi*, *A. echinites*, *A. lecanora*, *A. mauritana*, *H. grisea*, *H. hilla*, *H. Mexicana*, *H. moebi*, *H. nobilis*, *H. monacaria*, *H. forskali*, *H. edulis*, *H. axiloga*, *H. fuscocinerea* and *H. impatiens*	[12,36,44,67,72,79,93,96,97,98,99,100,101,102,103]
1259.5	1236	C_54_H_85_NaO_28_S	Holothurin A_3_	P	*H. lessoni*, *H. scabra*, *H. fuscocinerea* and *H. impatiens*	[12,44,69]
			Unidentified	N	*H. lessoni*	-
1287.6	1264	C_60_H_96_O_28_	Holothurinoside E_,_	P	*H. lessoni*, *H. forskali*	[12,77]
			Holothurinoside E_1_	P	*H. lessoni*, *H. forskali*	[12,77]
			Holothurinoside O	P	*H. lessoni*	[12]
			Holothurinoside P	P	*H. lessoni*	[12]
			17-dehydroxyholothurinoside A	P	*H. lessoni*, *H. grisea*, *B. cousteaui*	[4,12,104]
1301.6	1278	C_61_H_98_O_28_	Holothurinoside M	P	*H. lessoni*, *H. forskali*, *H. scabra*, *H. fuscocinerea* and *H. impatiens* *H. lessoni*	[12,67,69,70]
		C_60_H_94_O_29_	Unidentified	N		-
1303.6	1280	C_60_H_96_O_29_	Holothurinoside A	P	*H. lessoni*, *H. forskali*, *B. vitiensis*, *B. cousteaui*	[4,12,67,77,89]
			Holothurinoside A_1_	P	*H. lessoni*, *H. forskali*, *B. vitiensis*, *B. cousteaui*	[4,12,67,77,89]
			Holothurinoside Q	P	*H. lessoni*	[12]
			Holothurinoside S	P	*H. lessoni*	[12]
			Holothurinoside R	P	*H. lessoni*	[12]
			Holothurinoside R_1_	P	*H. lessoni*	[12]
1317.6	1294	C_61_H_98_O_29_	Holothurinoside N	P	*H. lessoni*, *H. forskali*	[12,67]
1475.6	1452	C_65_H_96_O_36_	Unidentified	N	*H. lessoni*	-
1477.7	1454	C_61_H_114_O_38_	Unidentified	N	*H. lessoni*	-
1479.7	1456	C_67_H_108_O_34_	Holothurinoside I	P	*H. lessoni*, *H. forskali*	[12,92]
1495.7	1472	C_61_H_116_O_39_	Holothurinoside K_1_	P	*B. subrubra*, *H. lessoni*	[12,59]
		C_72_H_112_O_31_	Unidentified	N	*H. lessoni*	-

^a^^*^: The composition was not measured through ESI analysis.

#### 2.1.1. Determination of the Saponin Structures by ESI-MS/MS

**Figure 2:**
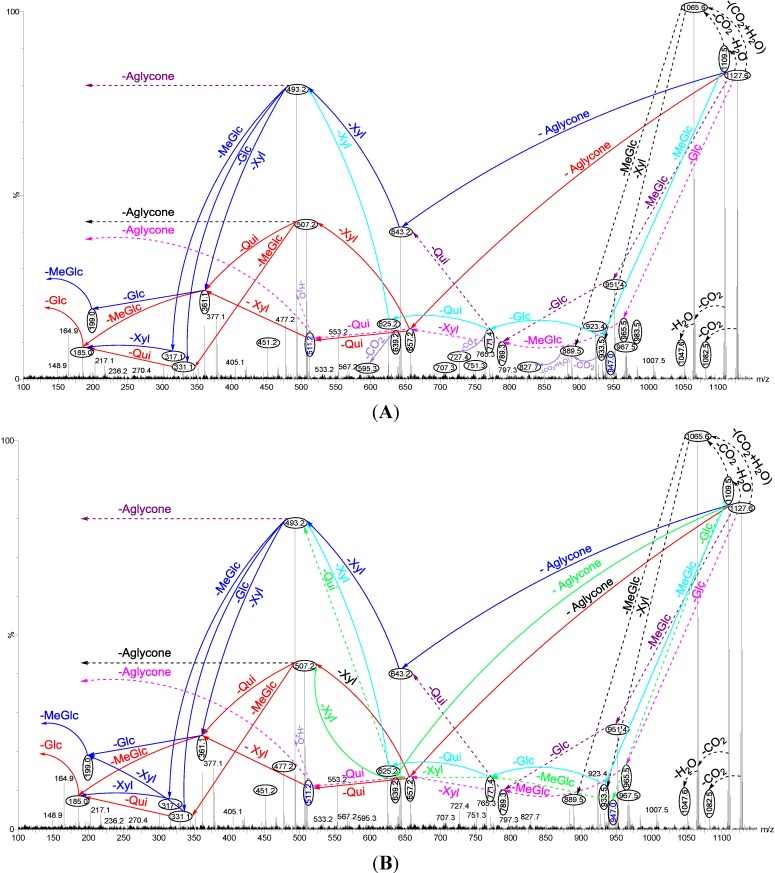
Positive tandem ESI spectrum analysis of saponins detected at *m/z* 1127.6, Fraction 17; (**A**) and Fraction 18; (**B**) Full and dotted arrows show the two main feasible fragmentation pathways. The figures indicate the collision-induced fragmentation of parent ions at *m/z* 1127.6. The consecutive losses of the aglycone (Agl), xylose (Xyl), quinovose (Qui) and 3-*O*-methylglucose (MeGlc) residues affords product ions detected at *m/z* 657.2, 507.2, 361.1 and 185.0, respectively, which indicate the structure of a novel saponin. The predominant peak (**A** and **B**) at *m/z* 493.2 corresponds to either the diagnostic sugar residue or the aglycone moiety. The major abundant peak (**A** and **B**) at *m/z* 507 also corresponds to either the key sugar residue or the aglycone moiety.

In order to differentiate between isomeric saponins following chromatographic separation, tandem mass spectrometry analysis was performed. Saponin ion peaks were analyzed by ESI MS/MS and confirmed using MALDI MS/MS. ESI-MS/MS was carried out using Collisional Induced-Dissociation (CID), creating ion fragments from the precursor ions. In general, the formation of fragments occurred predominantly by the cleavage of glycosidic bonds in the positive ion mode (Figure 2), which was applied to identify the structure of saponins. Interpretation and assignment of fragment ions of MS/MS spectra provided the key information for the structural elucidation of saponins. CID can provide valuable structural information about the nature of the carbohydrate residues, as it preferentially cleaves glycosides at glycosidic linkages, which makes assignment of the sugar residues and elucidation of the structure relatively straight forward.

ESI-MS was used to distinguish the isomeric saponins as described by Song *et al*. [87]. Following HPCPC separation, tandem mass spectrometry coupled with electrospray ionization (ESI-MS/MS) allowed the identification of isomers. MS/MS spectra of these ions provided detailed structural information and enabled differentiation of the isomeric saponins following HPCPC separation. The stepwise structure elucidation analysis applied to the ion at *m/z* 1127.6 (non-sulfated saponins), obtained from Fractions 17 and 18 is shown in Figure 2A,B. The peak at *m/z* 1127 was shown to contain at least three different saponin congeners.

**Figure 3:**
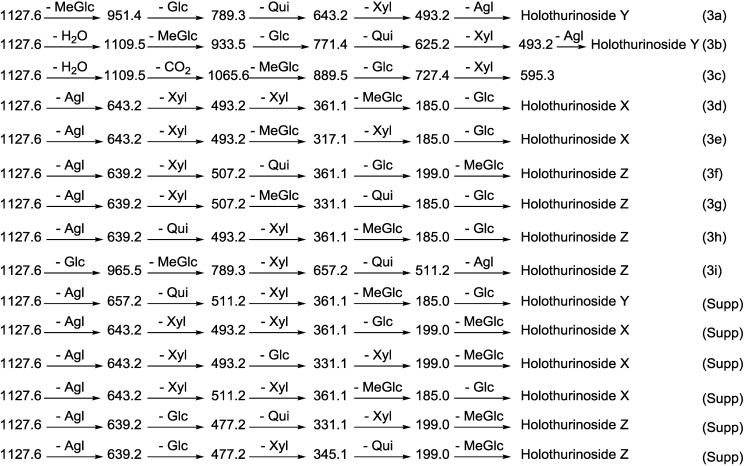
Schematic fragmentation patterns of the ion detected at *m/z* 1127.6.

CID initiates two feasible fragmentation pathways of cationized parent ions shown in full with dotted arrows. First, the successive losses of the sugar moieties 3-*O*-methylglucose (MeGlc), glucose (Glc), quinovose (Qui) and xylose (Xyl) followed by the aglycone (Agl) unit generate ion products detected at *m/z* 951.4, 789.3, 643.2, and 493.2, respectively (Figure 2A,B), which proposed the structure of Holothurinoside Y (Figure 3a). In another isomer, the sequential losses of MeGlc, Glc, Xyl, and Xyl followed by the aglycone residue correspond to ions observed at *m/z* 951.4, 789.3, 657.2 and 507.2, respectively, which postulates the structure of Holothurinoside X. In this case, the ions at *m/z* 493.2 and 507.2 correspond to the sodiated aglycone moieties.

Further, the consecutive losses of MeGlc, Glc, Qui, and Xyl from the ion at *m/z* 1109.5 generated the fragment ions shown in Figure 3b, confirming the structure of Holothurinoside Y. In addition to 1109.6, the ion at 1065.6 can be fragmented and produced ion products exhibited in Figure 3c. The sugar moiety was found to be identical to those of Intercedenside D and Eximisoside A isolated from sea cucumbers *Mensamaria intercedens* [105] and *Psolus eximius* [37], respectively. Both groups also stated the ions at *m/z* 625.2 and 493.1 correspond to [MeGlc + Xyl + Glc + Xyl + Na]^+^ and [MeGlc + Xyl + Glc + Na]^+^, respectively, which agrees with our results.

Secondly the decomposition of the precursor ions can also be triggered by the losses of the aglycone residues, creating peaks at *m/z* 657.2, 643.2 and 639.2 (Figure 2A,B) corresponding to the complete sugar moieties of the ion at *m/z* 1127.6, and indicates the presence of three types of aglycones. The consecutive losses of the aglycone (generated ion at *m/z* 657.2), Xyl (generated ion at *m/z* 507.2), Qui (generated ion at *m/z* 361.1), and MeGlc (generated ion at *m/z* 185.0), respectively, were produced by glycone and aglycone fingerprint peaks from the precursor ion, confirming the structure of Holothurinoside Y. In the second isomer, the consecutive losses of the aglycone, Xyl, Xyl and MeGlc followed by Glc presenting masses described in Figure 3d, additionally indicate that the decomposing ions were generated from the sodiated ion at *m/z* 1127.6. In this case, the ions at *m/z* 493.2 and 507.2 correspond to the key diagnostic sugar resides. Moreover, the ion at *m/z* 493.2 generated ions at *m/z* 317.1 and 185.0 by the losses of MeGlc and Xyl, respectively (Figure 3e), further confirm that the fragment ions unambiguously originate from a novel sodium-cationized saponin which we name as Holothurinoside X.

The ions at *m/z* 657.2 and 643.2 resulted from the loss of aglycones from the parent ion at *m/z* 1127.6, and are the fragment ions corresponding to the complete saccharide chains, which subsequently (Figure 2A) produce the ions at *m/z* 511.2 or 507.2 and ion at *m/z* 493.2 due to the losses of Qui or Xyl and Xyl residues. The ions (*m/z* 511) further fragmented to form ions of the same *m/z* value at *m/z* 361 and *m/z* 199 or 185. The observation of ions at *m/z* 507.2 and 511.2 further supports the above conclusion. The ion at *m/z* 511.2 can also be ascribed to the mass of the sodiated aglycone.

Similar analysis was carried out on another isomer (Figure 2B). As can be seen in the figure, the spectrum has (green arrows) a different fragmentation pattern from that seen in other isomeric compounds even though they have the same *m/z* value which indicates the presence of another isomer. For this isomer, the consecutive losses of the aglycone, Xyl, Qui and Glc or MeGlc units generated signals shown in Figure 3f, further confirmed the structure of one of the isomeric compounds (Figure 2B). Alternatively, the ion at 507.2 led to the ions at *m/z* 331.1 and 185.0 by the sequential loss of MeGlc and Qui (Figure 3g). On the other hand, the consecutive losses of the aglycone, Qui, Xyl and Glc or MeGlc units generated ion fingerprints illustrated in Figure 3h, which postulates the structure of another isomer from this compound (Holothurinoside Z). The decomposition of the parent ion can also be triggered by the loss of sugar moiety, namely Glc, MeGlc, Xyl, and Qui followed by the aglycone which generated the product ions indicated in Figure 3i. It should be noted that the major characteristic peaks at 493, 507 and 511 correspond to either the sodiated partial glycoside compositions or the sodiated aglycone moieties [12,59,77,106], which further confirmed the presence of isomeric saponins. The full analysis can be seen in Supplementary Figure S2.

The implementation of these molecular techniques on all ions detected in the MALDI/ESI spectra allows us to identify the molecular structures of the saponins. Key fragments from the tandem MS spectra of the positive ion mode of MALDI and ESI were reconstructed according to the example illustrated in order to propose the saponin structures. On the basis of these fragment signatures, the structures of three novel isomeric saponins have been elucidated. Some of these compounds share the diagnostic *m/z* 493 and/or 507, and *m/z* 639 and/or 657 key signals as a signature of the sodiated oligosaccharide residues (Table 2).

**Table 2:** Key diagnostic ions in the MS/MS of the holothurians saponins.

Diagnostic Ions in CID Spectra of [M + Na]^+^				
*m/z* signals (Da)				
	493	507	523	657
Chemical signatures	MeGlc-Glc-Xyl + Na	MeGlc-Glc-Qui + Na	MeGlc-Glc-Glc +Na	MeGlc-Glc-Qui-Xyl + Na

The structures of three isomeric saponins were ascribed to the ions detected at *m/z* 1127.6 (Figure 2A,B and Figure 3 and Supplementary Figure S2). The MS/MS spectra show the presence of three different aglycone structures, namely ions detected at *m/z* 657.2, 643.2 and 639.2 due to the losses of aglycone moieties. This analysis revealed the presence of at least three different isomers with different aglycone moieties *m/z* values of 470, 484 and 488 and differ in the lateral side as the MS/MS spectra generated the sodiated diagnostic fragments at *m/z* 493, 507 and 511, respectively. The structures of the aglycones were proposed based on the literature. The predominant fragment ion at *m/z* 493 corresponds to the sodium adduct sugar residue [MeGlc-Glc-Xyl + Na] side chain or the sodiated aglycone moiety. Similar conclusions were drawn by Zhang *et al.* [18] and Silchenko *et al*. [107] for triterpene glycosides.

**Figure 4:**
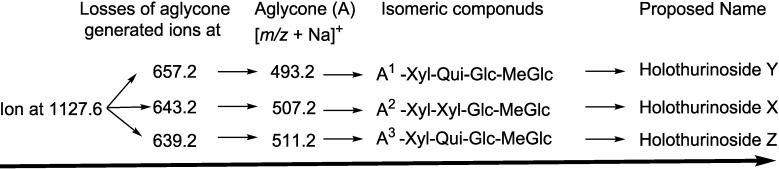
The schematic diagram of the proposed isomeric structures of ion at *m/z* 1127.6.

Based on the literature [12,59,69,77] and as the above analysis indicates, ions detected at *m/z* 657, and 639 correspond to the sodiated Xyl-Qui-Glc-MeGlc. The ion at 643 yields ions at 511 and 493 by the loss of Xyl. These ions are recognized as the key diagnostic fragments in triterpenoid saponins. These isomers were composed of four monosaccharides including MeGlc-Glc-(Xyl or Qui)-Xyl. The proposed structures are shown in Figure 4, which correspond to three novel saponins for which we propose the names Holothurinosides Y, X and Z.

The loss of aglycone with *m/z* value of 470 Da from the ions at *m/z* 1109.5, 1095.5, 1065.6, 951.4, 947.0, 889.5 and 618.1 generated ions that were detected at *m/z* 639.2, 625.2, 595.3, 481.2, 477.2, 419.1 and 148.9, respectively. Moreover, the loss of another aglycone (*m/z* value of 484 Da) from the ions at *m/z* 1109.5, 1051.6, 951.4, 935.5 and 889.5 generated ions of *m/z* 625.2, 567.2, 467.2, 451.2 and 405.1 respectively. In addition the loss of the third aglycone (*m/z* value of 488 Da) from the ions at *m/z* 1007.5, 947.0, 939.4, 933.5 and 691.4 generated ions that were observed at *m/z* 519.3, 459.2, 451.2, 445.3 and 203.0, respectively.

Tandem MS analysis revealed the presence of three different aglycones with *m/z* values of 470, 484 and 488 confirming the presence of the isomeric structures. The carbohydrate moieties of the isomeric saponins were found to be composed of four sugar units, which were identical to the sugar component of Holothurinoside C, Desholothurin A, Intercedenside D and Eximisoside A [12,37,105].

As can be seen in Figure 2 the abundance of signals for the ions at *m/z* 493, 507 and 511 is 8:4:1, respectively. Our findings demonstrated that the ions at *m/z* 493 and 507 are formed from the decomposition of two isomers separately. In contrast, the ion at *m/z* 511 has resulted from the disintegration of one isomer which can explain this discrepancy in intensity. The losses of H_2_O and CO_2_ or their combination results from cleavage at the glycosidic linkages as noted by Waller and Yamasaki [2]. The ions detected at *m/z* 1109.5, 1082.5 and 1065.6 resulted from the sequential losses of H_2_O, CO_2_ and the combination of H_2_O and CO_2_ from the parent ion. The first two fragments correspond to the sequential losses of water and carbon dioxide.

These two peaks at *m/z* 1127.6 and 1141.5 (Figure 1, Figure 2, Figure 3 and Table 1), were found to correspond to at least three and two isomers, respectively.

Kitagawa and associates [90] suggested the structure of the ion at *m/z* 1125.5, later named as Holothuriosides C/C_1_, based on their NMR and field desorption mass spectrum (FD-MS) data. Therefore, the structure of one of the isomers in the ion at *m/z* 1127.6 is produced due to reduced structure in the lateral chain. This is similar to what we see in the formation of Holothurinoside Y. Rodriguez and coworkers [89], however, demonstrated a different structure for the aglycone part of the ion observed at *m/z* 1125 compared to that reported by Kitagawa and co-workers.

**Figure 5:**
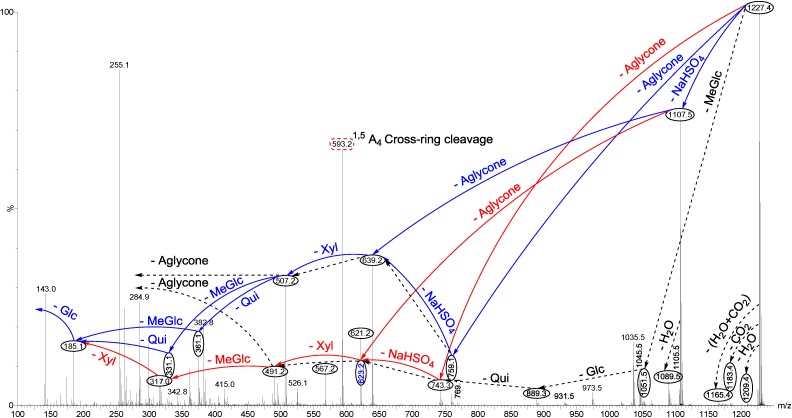
(+) ion mode ESI-MS/MS spectrum of sulfated saponins detected at 1227.4. Full and dotted arrows show the three main feasible fragmentation pathways. The consecutive losses of NaHSO_4_, Agl, Xyl, MeGlc and Qui residues affords product ions detected at *m/z* 1107.5, 639.0, 507.0, 331.1 and 185.0, respectively.

**Figure 6:**
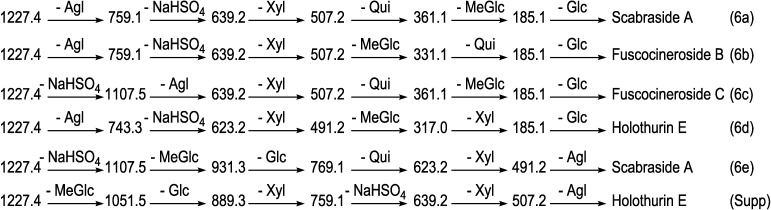
Schematic fragmentation patterns of the ion detected at *m/z* 1227.4.

The other example of an isomeric compound is the ion at *m/z* 1227.4. The structural elucidation of this ion using tandem MS is demonstrated in Figure 5. As can be seen in the MS/MS spectrum, the two ion peaks at *m/z* 743.3 and 759.1 correspond to the losses of different aglycone moieties with *m/z* values of 484 and 468, respectively, demonstrating the presence of isomeric compounds. Further, this MS/MS analysis revealed the presence of a sulfate group in the structure of the isomers. Similar to ions at *m/z* 1243.5 and 1259.5, after collisional activation, parent ions are subjected to three dissociation pathways shown using full and dotted arrows in Figure 5. First, the consecutive losses of the aglycone, sodium monohydrogen sulfate (NaHSO_4_), Xyl, Qui and MeGlc residues followed by Glc afford product ions that were described in Figure 5 and Figure 6a. Alternatively, the sequential losses of MeGlc and Qui from the key diagnostic peak (*m/z* 507.2) generated ions as shown in Figure 6b. Therefore, in this case, the ions at *m/z* 507.2 correspond to the sodiated key diagnostic sugar residue.

Secondly, the decomposition of the parent ion can also be triggered by the loss of sugar moiety, namely MeGlc, Glc, Qui, NaHSO_4_ and Xyl followed by the aglycone residue which generated daughter ions at *m/z* 1051.5, 889.3, 743.3, 623.2 and 491.2, respectively. In this case, the ions at *m/z* 491.2 correspond to the sodiated aglycone moiety (*m/z* value of 468).

Finally, the fragmentation of the parent ions can also be initiated with the loss of the sulfate group. The consecutive losses of NaHSO_4_ and the Agl units followed by the sequential losses of the sugar moiety, namely Xyl, Qui and MeGlc produced masses illustrated in Figure 5 and Figure 6c; ions at 639.2, corresponds to the total desulfated sugar moiety. This saponin possesses the common *m/z* 507.2 key signal as a fingerprint of MeGlc-Glc-Qui + Na^+^.

In another isomer, the consecutive losses of the aglycone, NaHSO_4_, Xyl and MeGlc residues generate product ions of *m/z* 743.3, 623.2, 491.2 and 317.0, respectively, and postulate the structure of a new isomer, which we propose to name Holothurin E (Figure 6d). In this case the ion at *m/z* 491.2 corresponds to the sodiated sugar residues. Further, the consecutive loss of NaHSO_4_ followed by the aglycone (*m/z* 484) and Xyl residue generated ions at *m/z* 1107.5, 623.2, and 491.2, respectively, confirming the structure of this isomer. Alternatively, the losses of NaHSO_4_ followed by the sugar moiety, namely MeGlc, Glc, Qui and Xyl followed by the aglycone unit generated daughter ions indicated in Figure 6e, confirming the structure of Scabraside A.

The loss of the aglycone (with *m/z* value of 468) from the ions at *m/z* 1107.5, 1089.5 and 1035.5 generated ions detected at *m/z* 639.2, 621.2 and 567.2, respectively. The loss of the aglycone (with *m/z* value of 484 Da) from the ions at *m/z* 1107.5, 1105.5 and 1051.5 generated ions observed at *m/z* 623.2, 621.2 and 567.2, respectively. The complete analysis can be seen in Supplementary Figure S3.

The losses of water and/or carbon dioxide were observed from the spectrum. For instance, the ions at *m/z* 1089.5 and 1045.5 were generated by the sequential losses of H_2_O and CO_2_, from the desulfated parent ions (*m/z* 1107.5), or the sequential losses of CO_2_ and H_2_O molecules from the parent ions generated ions at *m/z* 1183.5 and 1165.5, respectively.

The identification of compounds was confirmed by MS-MS analysis and was based on the published literature as shown in the last column of the Table 1. For the saponin detected at *m/z* 1227.4, the MS/MS spectrum exhibited the key diagnostic peaks at *m/z* 491, 507 and 639, which confirmed the presence of 24-dehydroechinoside A (synonymous with Scabraside A) and Fuscocinerosides B and C along with a novel isomeric saponin, Holothurin E. This finding was in agreement with those reported by Bondoc *et al.* [69]. They described this peak as corresponding to 24-dehydroechinoside A (synonymous with Scabraside A) or Fuscocinerosides B and C, or other isomers, differing only in the lateral side chain of their aglycone units. Cucumarioside H_3_ possesses the same molecular weight (*m/z* 1227) with those isomeric compounds [108], however, the structure of this compound was not detected in this species.

As Van Dyck *et al*. [59] also stated, the differences between isomeric saponins are not always measurable by applying MS methodology which only relies on low-kinetic energy CID. The saponin detected at *m/z* 1227.4 could correspond to either Fuscocineroside B or C, the two molecules differ only at the level of the lateral chain of their aglycones.

The MS/MS analysis of ions at 1227.4 revealed a very similar fingerprint profile with those reported for Holothurin A and Holothurin A_3_, which show the intrinsic relationship between these saponin congeners.

**Figure 7:**
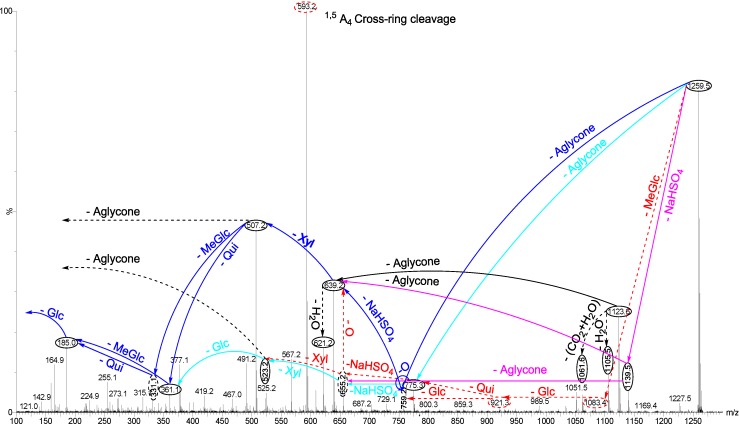
(+) ion mode ESI-MS/MS spectrum of saponins detected at *m/z* 1259.5 from Fraction 22. This spectrum shows the presence of two different aglycones indicating the presence of isomeric saponins. The consecutive losses of NaHSO_4_, Agl, Xyl, Qui and MeGlc followed by Glc residue affords product ions detected at *m/z* 1139.5, 639.2, 507.2, 361.1 and 185.0, respectively, which indicate the structure of Holothurin A_3_. Full and dotted arrows illustrate the three main possible fragmentation pathways.

**Figure 8:**
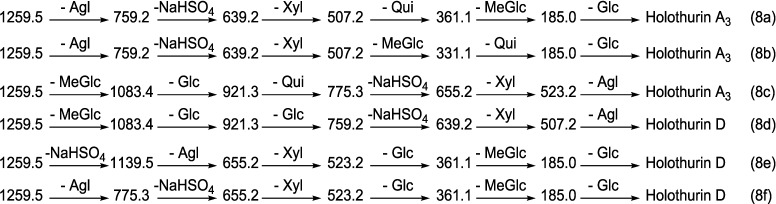
Schematic fragmentation patterns of the ion detected at *m/z* 1259.5.

Another typical chemical structure elucidation of isomeric saponins by tandem MS is exemplified in Figure 7. This spectrum shows the ion signature of the ion detected at *m/z* 1259.5 from Fraction 22 under tandem MS. Tandem MS analysis revealed the presence of two different aglycones with *m/z* values of 484 and 500, confirming the presence of chemical isomeric structures. Tandem MS analysis also distinguished the presence of a sulfate group in the structure of isomers. After collisional activation, *m/z* 1259.5 cations are subjected to three dissociation pathways shown using full and dotted arrows. First, as described in Figure 7, consecutive losses of aglycone, NaHSO_4_, Xyl, Qui and MeGlc residues generate the product ions demonstrated in Figure 8a and Figure S4 Supplementary Data. Alternatively, the sequential losses of MeGlc and Qui from the key diagnostic peak (*m/z* 507) generated ions shown in Figure 8b. Therefore, in this case the ion at *m/z* 507.2 corresponds to the key diagnostic sugar residue.

The decomposition of the parent ion can also be triggered by the loss of sugar moiety, namely MeGlc, Glc, Qui, NaHSO_4_ and Xyl followed by the aglycone which generated daughter ions illustrated in Figure 8c, which confirms the structure of Holothurin A_3_. It is clear that the ion at *m/z* 523.2 is a signature of the sodiated aglycone. The sequential losses of MeGlc, Glc, Glc, NaHSO_4_ and Xyl followed by the aglycone afford ions as shown in Figure 8d for which we propose the name Holothurin D. In this case, the ion at *m/z* 507.2 is the second most abundant fragment ion of the signature of the sodiated aglycone.

Alternatively, the fragmentation of the parent ions can also be initiated by the loss of the sulfate group. The consecutive losses of NaHSO_4_ and the aglycone unit followed by the sequential losses of the sugar moiety, namely Xyl, Glc and MeGlc produced the masses exhibited in Figure 7 and Figure 8e. The 655.2 ion corresponds to the total desulfated sugar moiety.

The reconstruction of fragment ions generated by tandem MS also identifies the existence of a novel isomeric saponin. The fragmentation of the precursor ion can also be initiated by the loss of aglycone moiety (Figure 7) which generated the mass at *m/z* 775.3 corresponding to the entire sulfated-sugar components. The continuous losses of the aglycone, NaHSO_4_, Xyl, Glc and MeGlc followed by Glc yielded the ion fragments shown in Figure 8f. In this case the ion at *m/z* 523.2 corresponded to the sodiated sugar residue and further confirmed the presence of a novel saponin (Holothurin D).

The loss of aglycone (with *m/z* value of 500) from the ions at *m/z* 1139.5, 1083.4, 967.2, 963.2, 947.4, 890.4, 877.4 and 847.3 generated ions of *m/z* 639.2, 583.1, 467.0, 463.0, 447.2, 390.9, 377.1, and 347.1, respectively. The loss of aglycone (with *m/z* value of 484 Da) from the ions at *m/z* 1139.5, 1123.6, 1105.5, 1051.5, 1033.6, 757.2, 729.1 and 639.2 generated ions observed at *m/z* 655.2, 639.2, 621.2, 567.2, 549.3, 273.1, 245.0 and 155.0, respectively. The loss of the aglycone from the ion at *m/z* 1123.6 yields the ion at *m/z* 639.2. Therefore, the ions at *m/z* 639.2 and subsequently 507.2 can be generated by the losses of both aglycones and the key diagnostic sugar unit which can explain the higher intensity of these peaks compared to the ions at 655.2 and 523.2 in the new isomeric saponin, which is produced by the loss of one type of aglycone.

**Figure 9:**
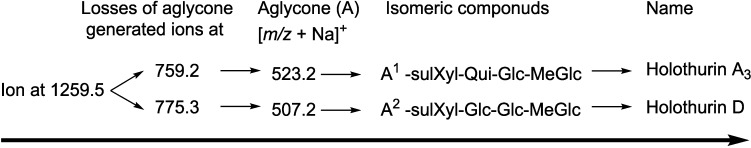
A schematic diagram of the proposed isomeric structures of ion at *m/z* 1259.5.

This analysis revealed the presence of at least two different isomers with diverse aglycone and sugar moieties. These isomers are also tetraglycosidic saponins. The proposed structures are shown in Figure 9, which corresponds to Holothurin A_3_ and a novel saponin; Holothurin D.

The structures of aglycones are similar to those reported for Holothurin A, Holothurin A_3_. The different fragmentation behaviors have been observed from the positive ESI-MS/MS spectra of saponins with *m/z* 1127.6. However, the tandem MS data of the carbohydrate moiety, in one isomer, was identical to those of the sugar component of Holothurin A, indicating the tetrasaccharide chain of Holothurin A_3_ was composed of 4*-O-*sulfated Xyl, Qui, Glc and MeGlc residues with the ratio of 1:1:1:1. Further the spectrum was similar to the spectrum of Holothurin A, the signals were coincident with those of Holothurin A.

The molecular analysis revealed a series of ions consistent with the presence of Holothurin A_3_ with an elemental composition of C_54_H_85_NaO_28_S in addition to one novel saponin. The observed fragments are consistent with the structure of the Holothurin A_3_ proposed by Dang and coworkers [44]. Our data are in agreement with ESI-MS data obtained by Dang *et al*, identified as the 3β, 12α, 17α, 25-tetrahydroxyholost-9(11)-ene-22-one 3-*O*-[(3-*O*-methyl)-β-d-glucopyranosyl-(1→3)-β-d-glucopyranosyl-(1→4)-β-d-quinovopyranosyl-(1→2)-(4-*O*-sulfo)-β-d-xylopyranoside] sodium salt.

The data indicate that the terminal sugar is the first moiety to be lost under CID. Since these isomers (A_3_ and D) contain the same terminal sugar units in their sugar residue, they yielded the ions with the same *m/z* values (*m/z* 361 and 185).

### 2.2. Key Diagnostic Fragments in the Sea Cucumber Saponins

The common key fragments facilitated the structure elucidation of novel and reported saponins. Tandem mass spectrometry analysis of saponins revealed the presence of several diagnostic key fragments corresponding to characteristic structural element of saponins as summarized in Table 2. Here we report a new diagnostic key fragment at *m/z* 493 corresponding to either the aglycone moiety or the sugar residue.

The structures of saponins were elucidated by the identification and implementation of the key fragment ions produced by tandem mass spectrometry. The presence of the oligosaccharide residue (*m/z* 493 and/or 507 and/or 511 and/or 523 and/or 657) simplified the determination of the saponin structure.

The ESI analysis revealed that the ion *m/z* 1259.5 is an isomeric compound that corresponded to a new saponin (Holothurin D) and Holothurin A_3_, which was found in several species of sea cucumbers.

**Figure 10:**
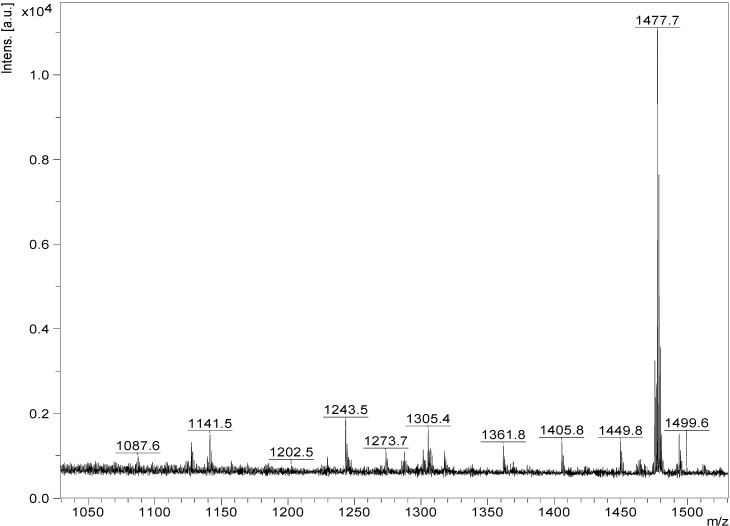
The MALDI mass spectrum of HPCPC Fraction 18 from the viscera of the *H. lessoni* in the (+) ion mode. A mass range of 1000 to 1500 Da is shown here.

MALDI-MS analysis was also performed on the isolated saponins. The prominence of the parent ions [M + Na]^+^ in MS spectra also enabled the analysis of saponins in mixtures or fractions. As a representative example, the MALDI mass spectrum (between 1000 and 1500 Da) of the saponin extract obtained from the HPCPC Fraction 18 is shown in Figure 10.

The peaks at *m/z* 1125.5 (Holothurinosides C/C_1_), 1141.5 (Desholothurins A/A_1_), 1157.5 (Holothurinoside J_1_), 1227.4 (Fuscocinerosides B/C or Scabraside A and a novel isomer), 1243.5 (Holothurin A), 1287.6 (Holothurinosides E/E_1_/O/P), 1301.6 (Holothurinoside M), 1303.6 (Holothurinosides A/A_1_/Q/R/R_1_/S), 1317.6 (Holothurinoside N) and 1495.7 (Holothurinoside K_1_) represent known compounds whereas the following peaks represent novel saponins 1087.6, 1111.5, 1123.5, 1127.6, 1305.4, 1361.8, 1405.8, 1417.7, 1449.8 1475.7, 1477.7 and 1493.7 is shown in Table 1.

### 2.3. MALDI-MS/MS Analysis of Saponins in Positive Ion Mode

Saponin ion peaks were further analyzed by MALDI MS/MS and reconfirmed the ESI results. The MALDI MS/MS figures can be found in Supplementary Figure S4 and Figure S5. The techniques used are able to distinguish the structural differences among the isomers following HPCPC separation. This analysis also confirmed the presence of saponins reported in the literature and allowed the discovery of new saponin congeners. As a typical example, the MALDI-MS/MS mass spectrum for the ion detected at *m/z* 1259.5 (sulfated triterpene glycoside) is shown in Supplementary Figure S4. The fragmentation pattern of the sodiated compound at *m/z* 1259.5 from fraction 20 in successive MS experiments is discussed in detail below for stepwise elucidation of the molecular structure of these compounds.

CID activates three feasible fragmentation pathways of cationized parent ions shown in full and dotted arrows. First, the continuous losses of Agl, NaHSO_4_, Xyl, Qui and MeGlc residues yielded to ion fragments described in Figure 8a. In this case the ion at *m/z* 507.2 corresponds to the sugar reside [MeGlc-Glc-Qui + Na]^+^.

Secondly, the decomposition of the parent ion can also be triggered by the sequential loss of a sugar moiety, namely MeGlc, Glc, Qui, NaHSO_4_ and Xyl followed by the aglycone which generated the daughter ions demonstrated in Figure 8c. This sequence of fragmentation corresponds to the known structure of Holothurin A_3_. For the second isomer, the consecutive losses of MeGlc, Glc, Glc, NaHSO_4_ and Xyl followed by the aglycone produced ions corresponding to the structure of Holothurin D (Figure 8d).

The fragmentation of the parent ions can also be initiated by the loss of the sulfate group. Then consecutive losses of NaHSO_4_ and the aglycone unit is followed by the sequential losses of the sugar moiety produced signals observed at *m/z* 1139.5 and 639.2 (Supplementary Figure S4); the latter peak corresponding to the entire desulfated sugar moiety; 639.2 [M + Na − 120 − 500 (aglycone)]^+^ and 507 [M + Na − 120 − 500 − 132 (Xly)]^+^. In addition, the sequential losses of the NaHSO_4_ (*m/z* 1139.2), Agl (*m/z* 655.2), Xyl (*m/z* 523.2), Glc (*m/z* 361.1) and MeGlc (*m/z* 185.0) support the isomer being Holothurin D (Figure 8e).

Holothurin A_3_ was originally isolated from the methanol extract of the sea cucumber *H. scabra* by Dang *et al.* [44]. This group indicated Holothurins A_3_ as a sulfated tetrasaccharide triterpene glycoside, contacting sulXyl, Qui, Glc and MeGlc with the ratio of 1:1:1:1. Here we describe the structure of a novel isomer, Holothurin D, with the same *m/z* value but different aglycone and sugar residues. The structure of this sulfated tetrasaccharide triterpene comprised of sulXyl, Glc and MeGlc with the ratio of 1:2:1, respectively.

Peaks formed by the losses of water (18 Da) and/or carbon dioxide (44 Da) have been annotated in Supplementary Figure S4. For instance, the peaks observed at *m/z* 1241.5 and 1223.5 were generated by the sequential losses of two H_2_O molecules from the parent ion and the major ion at *m/z* 621.2 corresponds to the loss of water from *m/z* 639.2. This peak could also be generated by the loss of aglycone from the ion at *m/z* 1105.5.

**Figure 11:**
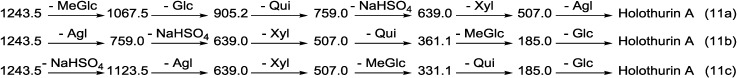
Schematic fragmentation patterns of the ion detected at *m/z* 1243.5, Holothurin A.

Another typical chemical structure elucidation of sulfated saponins by tandem MS is exemplified by the ion detected at *m/z* 1243.5 in Supplementary Figure S5. CID induces three feasible fragmentation pathways of the cationized sulfated parent ions like ion at *m/z* 1259.5 shown in full and dotted arrows. First, the loss of the sugar unit; the successive losses of MeGlc, Glc, Qui, sulfate and Xyl followed by the aglycone unit generate the ions described in Figure 11a. The fragmentation masses correspond to those which would be generated by Holothurin A. The ion at *m/z* 507.0 corresponds to the aglycone moiety.

Secondly, the decomposition of the precursor ions can also be triggered by the loss of the aglycone residue, creating peaks at *m/z* 759.0 (Figure 11b) corresponding to the sulfated sugar moiety. The consecutive losses of the Agl, NaHSO_4_, Xyl, Qui and MeGlc units generate the masses shown in Figure 11b. The third viable pathway is elicited by the loss of sulfate group. The consecutive losses of NaHSO_4_ and the Agl unit producing signals observed at *m/z* 1123.5 and 639.0, the latter peak corresponding to the complete desulfated sugar moiety exhibited in Figure 11c. All these possible fragmentation routes are consistent with ions corresponding to the fragmentation of sodiated Holothurin A (*m/z* 1243.5).

The loss of 18 Da from the sodiated molecular ion, suggests the elimination of a neutral molecule (H_2_O) from the sugar group [24]. A similar predominant peak at *m/z* 593.2 was noted and the assignment as previously given was confirmed [12].

This MS/MS spectrum allows us to reconstruct the collision-induced fragmentation pattern of the parent ion (Figure 10) and consequently to confirm that ions monitored at *m/z* 1243.5 correspond to the Holothurin A [12,59,89,96].

Mass spectrometry provides a relatively swift and straightforward characterization of the elemental composition, saponin structure and distribution by the presence of the key ions at *m/z* 493, 507, 523 and 639 in the tandem spectra of the viscera extracts as described in detail in our previous publication [12].

**Figure 12:**
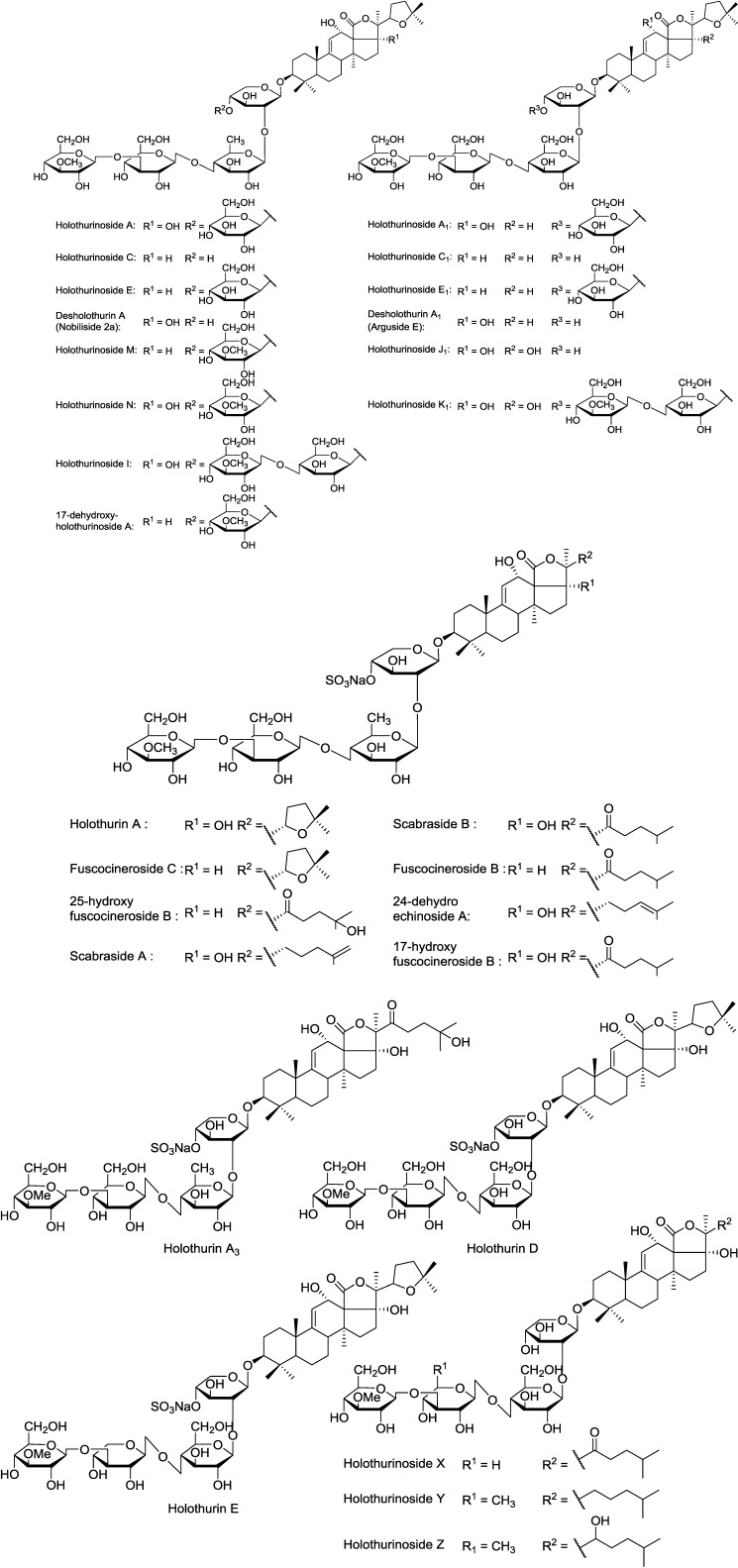
The structure of identified saponins in the viscera of *H. lessoni*. Holothurins D/E and Holothurinosides X/Y/Z are the novel compounds described in this paper.

Based on the MS/MS data and mass accuracy, saponins were categorized into seven distinct carbohydrate structural types [12]. In general, non-sulfated saponins are more conjugated with glycosides compared to sulfated saponins. Non-sulfated saponins had one to six monosaccharide units and six distinct structural types [12]. All sulfated saponins ranging from *m/z* 889 to 1259 had the structure [(MeGlc-Glc)-Qui-sulXyl-Aglycone] in which Xyl was sulfated. However, in some cases the sulfation of MeGlc and Glc has also been reported [20]. The chemical structures of the identified compounds are illustrated in Figure 12.

When comparing the MS/MS spectra of 1127.6 and 1259.5 (Figure 2 and Figure 7), it is notable that the *m/z* 523 fragment (the aglycone loss) of the [M + Na]^+^ ions observed with 1259.5 only, which corresponds to the presence of a new aglycone unit at *m/z* 500 (sodiated 523). Individual patterns were detected from sulfated and non-sulfated saponins as indicated in Holothurin A_3_ and Holothurinoside Y as representative examples. The main difference between both MS/MS spectra was the presence of a sulfate group in Holothurin A_3_.

The presence of a sulfate group is also confirmed for the ion at *m/z* 1227.4. The loss of 120 Da from the parent ion is the signature of a sulfate group in the saponin. On the other hand, both MS/MS spectra (compared to 1259.5) share the common *m/z* 507 key signal as a signature of the sodiated MeGlc-Glc-Qui oligosaccharide chain.

By comparison of the molecular weights and structures of both saponins (Holothurin A_3_, Holothurin A), they revealed mass differences between each other, such as 16 Da (*O*) mass differences between the aglycone of this saponin and Holothurin A, reflecting the small structural alterations and the intrinsic connections between them. Their MS/MS analysis indicated the presence of identical sugar moieties in both ions.

In some cases this methodology was not sufficiently precise to identify the molecular structure with certainty and several possibilities were offered by the literature. This paper is the first to describe the structure of these saponin congeners in this species and the first to show the MS/MS spectra of the ions at *m/z* 1127.6, 1227.4 and 1259.5 and assigned their fragmentation pattern. This sequential decomposition confirms the proposed structures Fuscocinerosides B/C, Scabraside A, Holothurins A/A_3_/D/E and Holothurinosides X/Y/Z.

The predominant fragment signal at *m/z* 593.2 results from α ^1,5^A_4_ cross-ring cleavage of the sulXyl residue which was consistent with previous findings for the MS/MS of sea cucumber saponins [12]. However, this peak was only detected as an intense signal in the sulfated saponins such as Scabraside A and Holothurin A_3_ whereas it was not observed in the non-sulfated saponins such as 1127.6. Therefore, the occurrence of this cross-ring cleavage appears to occur only with the sulfated Xyl (sulXyl). Moreover, this data confirms that cross-ring cleavages are more frequent in sulfated saponins. This phenomenon may occur as a result of the loss of sulfate group creating double bound in the Xyl residue. The intensity of the ion at *m/z* 593.2 resulted from the cross-ring cleavage is higher in the ESI-MS/MS spectra compared to those in the MALDI MS/MS spectra which might be consequence of the ESI CID. As highlighted there are three feasible MS/MS fragmentation pathways for the sulfated compounds, while there are only two possible fragmentation pathways for non-sulfated saponins.

Peaks corresponding to the natriation of complete sugar side chains were often quite intense in the product ion spectra of the natriated saponin precursor. Tandem mass spectra of these saponins reflected the multiple fingerprints with a range of relative intensities.

## 3. Experimental Section

### 3.1. Sea Cucumber Sample

Twenty sea cucumber samples of *Holothuria lessoni* Massin *et al.*, 2009, commonly known as Golden sandfish were collected off near Lizard Island (latitude; 14°41′29.46″ S, longitude; 145°26′23.33″ E), Queensland, Australia in September 2010 as described in [12]. The viscera (all internal organs) were separated from the body wall and kept separately in zip-lock plastic bags which were snap-frozen, then transferred to the laboratory and kept at −20 °C until use.

### 3.2. Extraction Protocol

The debris and sand particles were separated from the viscera manually and the visceral mass was freeze dried (VirTis, BenchTop K, New York, NY, USA). The dried specimens were then pulverized to a fine powder using liquid nitrogen and a mortar and pestle.

All aqueous solutions were prepared with ultrapure water generated by a Milli-Q water purification system (18.2 MΩ, Millipore, Bedford, MA, USA). All organic solvents were purchased from Merck (Darmstadt, Germany) except when the supplier was mentioned, and were either of HPLC grade or the highest degree of purity.

#### 3.2.1. Extraction of Saponins

The saponins were extracted as described previously [12]. The pulverized viscera sample (40 g) was extracted four times with 70% ethanol (EtOH) (400 mL) followed by filtration through Whatman filter paper (No.1, Whatman Ltd., Maidstone, England, UK) at room temperature. The extract was concentrated under reduced pressure at 30 °C using a rotary evaporator (Büchi AG, Flawil, Switzerland) to remove the ethanol, and the residual sample was freeze dried. The dried residue was successively extracted using a modified Kupchan partition procedure [109]: The dried extract (15 g) was dissolved in 90% aqueous methanol (MeOH), and partitioned against 400 mL of n-hexane (v/v) twice. The water content of the hydromethanolic phase was then adjusted to 20% (v/v) and then to 40% (v/v) and the solutions partitioned against CH_2_Cl_2_ (450 mL) and CHCl_3_ (350 mL), respectively. The hydromethanolic phase was concentrated to dryness using a rotary evaporator and freeze drier. The dried powder was solubilized in 10 mL of MilliQ water (the aqueous extract) in readiness for undergo chromatographic purification.

### 3.3. Purification of the Extract

The aqueous extract was placed on a prewashed Amberlite^®^ XAD-4 column (250 g XAD-4 resin 20–60 mesh; Sigma-Aldrich, MO, USA; 4 × 30 cm column) chromatography. After washing the column extensively with water (1 L), the saponins were eluted sequentially with MeOH (450 mL), acetone (350 mL) and water (250 mL). The eluates (methanolic, acetone and water fractions) were concentrated, dried, and redissolved in 5 mL of MilliQ water. Finally, the aqueous extract was partitioned with 5 mL isobutanol (v/v). The isobutanolic saponin-enriched fraction was either stored for subsequent mass spectrometry analysis or concentrated to dryness and the components of the extract were further examined by HPCPC and RP-HPLC. The profile of fractions was also monitored by Thin Layer Chromatography (TLC) using the lower phase of CHCl_3_:MeOH:H_2_O (7:13:8 v/v/v) solvent system.

### 3.4. Thin Layer Chromatography (TLC)

Samples were dissolved in 90% or 50% aqueous MeOH and 10 microliters were loaded onto silica gel 60 F_254_ aluminum sheets (Merck # 1.05554.0001, Darmstadt, Germany) and developed with the lower phase of CHCl_3_–MeOH–H_2_O (7:13:8) biphasic solvent system. The profile of separated compounds on the TLC plate was visualized by UV light and by spraying with a 15% sulfuric acid in EtOH solution and heating for 15 min at 110 °C until maroon-dark purple spots developed.

### 3.5. High Performance Centrifugal Partition Chromatography (HPCPC or CPC)

The solvent system containing CHCl_3_:MeOH:H_2_O- 0.1% HCO_2_H (7:13:8) was mixed vigorously using a separating funnel and allowed to reach hydrostatic equilibration. Following the separation of the two- immiscible phase solvent systems, both phases were degassed using a sonicator-degasser (Soniclean Pty Ltd. Adelaide, SA Australia). Then the rotor column of HPCPC™, CPC240 (Ever Seiko Corporation, Tokyo, Japan) was filled with the liquid stationary phase at a flow rate of 5 mL/min^−^^1^ by Dual Pump model 214 (Tokyo, Japan).

The CPC was loaded with the aqueous upper phase of the solvent system in the descending mode at a flow rate of 5 mL/min^−^^1^ with a revolution speed of 300 rpm. The lower mobile phase was pumped in the descending mode at a flow rate of 1.2 mL/min^−^^1^ with a rotation speed of 900 rpm within 2 h. One hundred and twenty milligrams of isobutanol- enriched saponins mixture was dissolved in 10 mL of the upper phase and lower phase in a ratio of 1:1 and injected to the machine from the head-end direction (descending mode) following hydrostatic equilibration of the two phases indicated by a clear mobile phase eluting at the tail outlet. This indicated that elution of the stationary phase had stopped and the back pressure was constant. The chromatogram was developed at 254 nm for 3.0 h at 1.2 mL/min^−^^1^ and 900 rpm using the Variable Wavelength UV-VIS Detector S-3702 (Soma optics, Ltd. Tokyo, Japan) and chart recorder (Ross Recorders, Model 202, Topac Inc. Cohasset, MA, USA). The fractions were collected in 3 mL/tubes using a Fraction collector. The elution of the sample with the lower organic phase proceeded to remove the compounds with low polarity from the sample within 200 mL of which several peaks were eluted. At this point (Fraction 54), the elution mode was switched to ascending mode and the aqueous upper phase was pumped at the same flow rate for 3.0 h. Recovery of saponins was achieved by changing the elution mode to the aqueous phase which allowed the elution of the remaining compounds with high polarity in the stationary phase. A few minor peaks were also monitored. Fractions were analyzed by TLC using the lower phase of CHCl_3_:MeOH:H_2_O (7:13:8) as the developing system. The monitoring of the fractions is necessary as most of the saponins were not detected by UV due to the lack of a chromophore structure. Fractions were concentrated with nitrogen gas.

### 3.6. Mass Spectrometry

The resultant HPCPC purified polar samples were further analyzed by MALDI and ESI MS to elucidate and characterize the molecular structures of compounds.

#### 3.6.1. MALDI MS

MALDI analysis was performed on a Bruker Autoflex III Smartbeam (Bruker Daltonik, Bremen, Germany). The laser (355 nm) had a repetition rate of 200 Hz and operated in the positive reflectron ion mode for MS data over the mass range of 400 to 2200 Da under the control of the Flexcontrol (V 3.3 build 108, Bruker Daltonik, Bremen, Germany). External calibration was performed using PEG. MS spectra were processed in FlexAnalysis (version 3.3, Bruker Daltonik, Bremen, Germany). MALDI MS/MS spectra were obtained using the LIFT mode of the Bruker Autoflex III with the aid of CID (Bruker Daltonik, Bremen, Germany). The isolated ions were submitted to collision against argon in the collision cell to collisionally activate and fragment, and afford intense product ion signals. For MALDI, a laser energy was used that provided both good signal levels and mass resolution, the laser energy for MS/MS analysis was generally 25% higher than for MS analysis.

The samples were placed onto a MALDI stainless steel MPT Anchorchip TM 600/384 target plate (Bruker Daltonik, Bremen, Germany). Alpha-cyano-4-hydroxycinnamic acid (CHCA) in acetone/iso-propanol in ratio of 2:1 (15 mg/mL) was used as a matrix to produce gas-phase ions. The matrix solution (1 µL) was spotted onto the MALDI target plate and air-dried. Subsequently 1 μL of sample was added to the matrix crystals and air dried. Finally, 1 μL of NaI (Sigma-Aldrich # 383112, St Louis, MI, USA) solution (2 mg/mL in acetonitrile) was applied onto the sample spots. The samples were mixed on the probe surface and dried prior to analysis.

#### 3.6.2. ESI MS

The ESI mass spectra were obtained with a Waters Synapt HDMS (Waters, Manchester, UK). Mass spectra were obtained in the positive ion mode with a capillary voltage of 3.0 kV and a sampling cone voltage of 100 V.

The other conditions were as follows: extraction cone voltage, 4.0 V; ion source temperature, 80 °C; desolvation temperature, 350 °C; desolvation gas flow rate, 500 L/h. Data acquisition was carried out using Waters MassLynx (V4.1, Waters Corporation, Milford, USA). Positive ion mass spectra were acquired in the V resolution mode over a mass range of 100–2000 *m/z* using continuum mode acquisition. Mass calibration was performed by infusing sodium iodide solution (2 μg/μL, 1:1 (v/v) water:isopropanol). For accurate mass analysis a lock mass signal from the sodium attached molecular ion of Raffinose (*m/z* 527.1588) was used.

MS/MS spectra were obtained by mass selection of the ion of interest using the quadrupole, fragmentation in the trap cell where argon was used as collision gas. Typical collision energy (Trap) was 50.0 V. Samples were infused at a flow rate of 5 μL/min, if dilution of the sample was required then acetonitrile was used [87]. Chemical structures were determined from fragmentation schemes calculated on tandem mass spectra and from the literature.

## 4. Conclusions

Marine invertebrates synthesize a plethora of small fascinating molecules with interesting chemical structures and potent biological properties. Holothurians are one class of marine invertebrate animals which are an important source of human food and traditional medicine, especially in some parts of Asia. In the past three decades, the scientific literature from several countries revealed that triterpene glycosides from sea cucumbers have a wide spectrum of biological effects.

The extract of the viscera of sea cucumber *H. lessoni* has been processed by applying HPCPC to purify the saponin mixture and to isolate saponin congeners and isomeric saponins. Other research groups have applied nuclear magnetic resonance (NMR) spectroscopy to obtain extensive structural information for saponins, but high-quantities of high-purity samples are usually required. In particular when the NMR signals are overlapping, the assignment of data is labor-intensive and very time consuming. Moreover, the measurement of the absolute configuration of the sugar moieties of a saponin cannot be completely solved by NMR methods alone [110]. Matrix-assisted laser desorption/ionization mass spectrometry (MALDI- MS) and electrospray ionization mass spectrometry (ESI-MS) techniques have become the preferred techniques for analysis of saponins. Mass spectrometry provides a highly sensitive platform for the analysis of saponin structures by generating product ions by the cleavage of the glycosidic bond.

The tandem MS approach enabled us to determine the structure of a range of saponins. The purity of HPCPC fractions allowed mass spectrometry analysis to reveal the structure of isomeric compounds containing different aglycones and/or sugar residues. Several novel saponins, along with known compounds were identified from the viscera of sea cucumber. We performed tandem mass spectrometry analysis on both sulfated and non-sulfated compounds, and compared their fragmentation profiles.

Our results highlight that there are a large number of novel saponins in the viscera indicating the viscera as a major source of these compounds. This paper is the first not only to deduce the structure of several novel isomeric saponins, including ions at *m/z* 1127 (Holothurinosides X/Y/Z), 1227 (Holothurin E, Scabraside A) and 1259 (Holothurins D/A_3_) in the viscera of *H. lessoni* but also to demonstrate the MS/MS profiles of the number of known triterpene glycoside congeners such as Fuscocinerosides B/C or Scabraside A and Holthurin A_3_. Peak intensities of fragment ions in MS/MS spectra were also correlated with structural features and fragmentation preferences of the investigated saponins; therefore, we were able to estimate the proportion of each isomer in the isomeric compounds. Evidence from tandem mass spectrometry suggested that the most abundant ions are generally attributed to the losses of aglycones and/or the key diagnostic sugar moieties (493, 507, 523, 639 and 643). Our results also reconfirmed the incidence of cross-ring cleavages to be higher in the sulfated compounds compared to non-sulfated glycosides. It is likely that the loss of sulfate group in the sugar moiety of saponins made them more susceptible for cross-ring cleavages.

For now, MS is one of the most sensitive and straightforward techniques of molecular analysis to determine saponin structure. This methodology of molecular structure identification using fragmentation patterns acquired from MS/MS measurements helps to propose and identify the structure of the saponins. It was found that under CID some of the identified saponins have the same ion fingerprints for their sugar units, yielding the same *m/z* product ions. Some of these saponins were easily characterized since their MS/MS spectra shared common fragmentation patterns for the key diagnostic signals at *m/z* 493 and/or 507 and 523, in addition to the vital peaks at *m/z* 639 and 657, corresponding to the oligosaccharide chain [MeGlc-Glc-Qui-Xyl + Na^+^]. We used mass spectrometry to evaluate the isomeric heterogeneity of their precursor ions as well as the structurally informative product ions.

Our finding indicates that the viscera are rich in saponins, in both diversity and quantity, and therefore their localization in the viscera is apparently related to the use of these organs in the defense against potential predators.

The chromatography techniques that were used in this study were able to separate saponins and isomeric saponins from sea cucumber to a purity which permits the structure elucidation of isomeric saponin congeners.

These novel saponins (Holothurinosides X/Y/Z and Holothurins E/D/) have great potential to be used for functional food ingredients (tonic foods), dietary supplements, food additives, food preservatives (because of emulsifying and foaming properties) and development of high value products for various industrial applications. For instance, they can be used as nutritional supplements or functional foods for human and animals. Therefore, this marine invertebrate is a valuable source for functional food.

This manuscript described the structure elucidation of seven reported compounds, Holothurin A or Scabraside B, 17-dehydroxy-holothurinoside A, Fuscocinerosides B/C or Scabraside A, 24-dehydroechinoside A and Holothurin A_3_ before obtaining the structure of five novel compounds, Holothurins D/E and Holothurinosides X/Y/Z.

This study confirms the viscera of *H. lessoni* as a source of saponins with a wide spectrum of structural diversity, including both novel sulfated and non-sulfated congeners. Our findings demonstrate that the study of new sea cucumber species has provided a large number of compounds that may have application as nutraceuticals, pharmaceuticals, agrochemicals, cosmeceuticals or as research reagents.

## Supplementary Files

Supplementary File 1
