# Supplementary material for: Structural Elucidation of Novel Saponins in the Sea Cucumber Holothuria lessoni
Source: Mar Drugs. 2014 Aug 8;12(8):4439–73. doi: 10.3390/md12084439 (PMC4145325; doi:10.3390/md12084439)
Supplement: Supplementary File 1 [file marinedrugs-12-04439-s001.pdf]

Supplementary Information

Figure S1. The thin-layer chromatography (TLC) pattern of the high performance centrifugal partition chromatography (HCPCP) fractions from the purified extracts of the viscera of the *Holothuria lessoni* sea cucumber using the lower phase of $CHCl_3$–MeOH–H_2O (7:13:8) system. The numbers under each lane indicate the fraction number in the fraction collector. The fractions 66 to 82 of one analysis (of 110 fractions) were pooled into either fraction 17, 18 or 19 as shown in Figure S1. TLC plates were sprayed with 15% sulfuric acid in ethanol to identify the saponin species with a maroon-dark purple color.

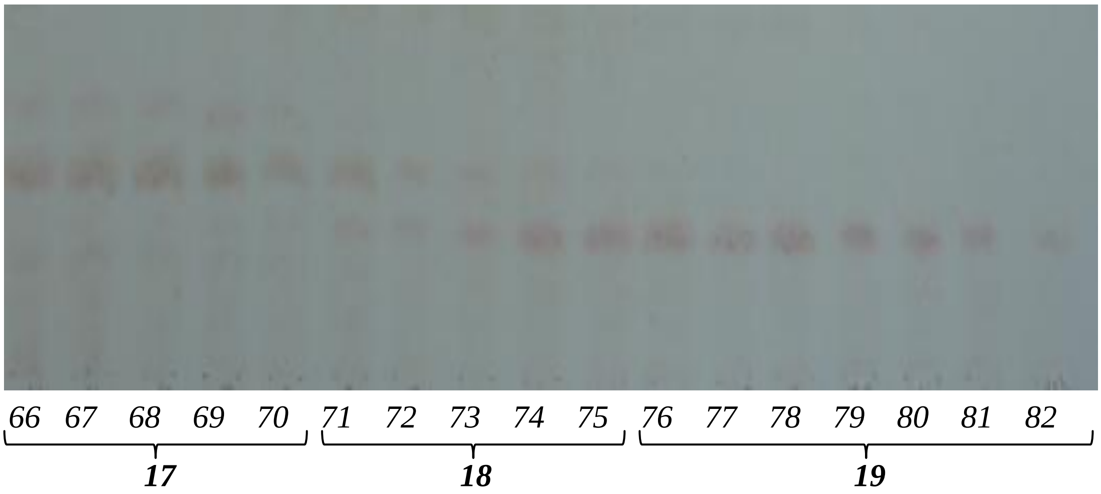

Figure S2. Ion fragmentation and proposed structures for the isomeric saponins of the precursor ion at *m/z* 1127.6, losses of neutral molecules (CO_2 and H_2O) (**a**); complete ESI-MS/MS fragmentation profile (**b**).

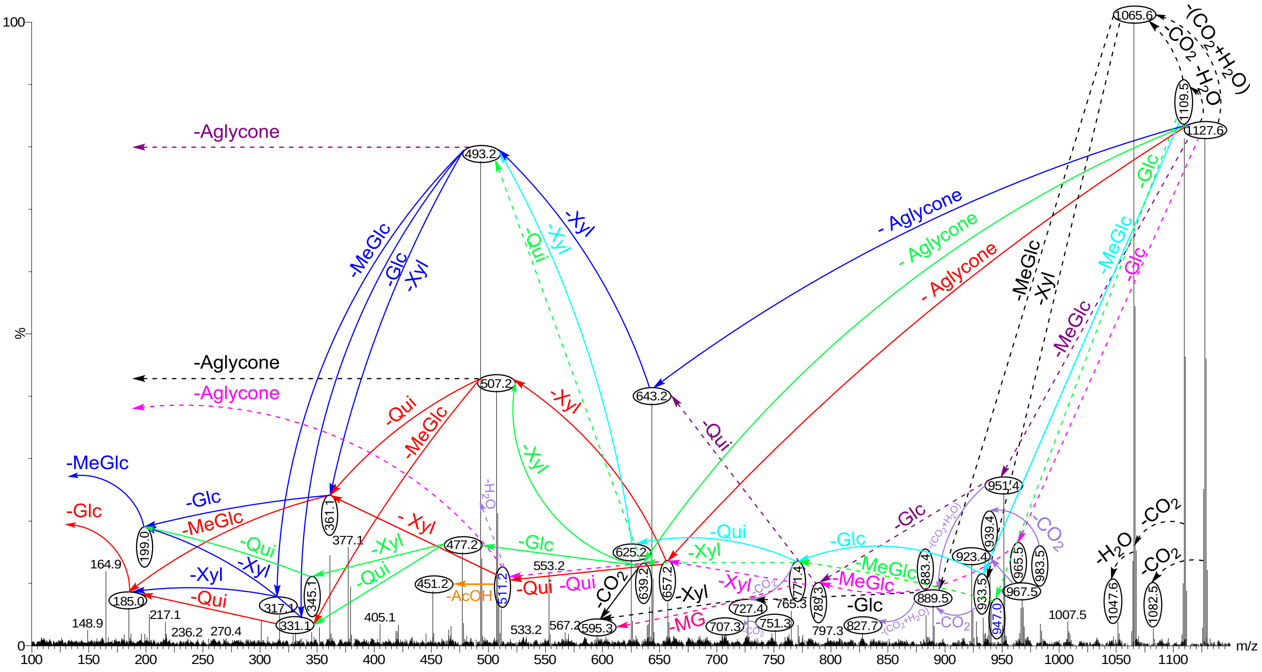

(a)

Figure S2. *Cont.*

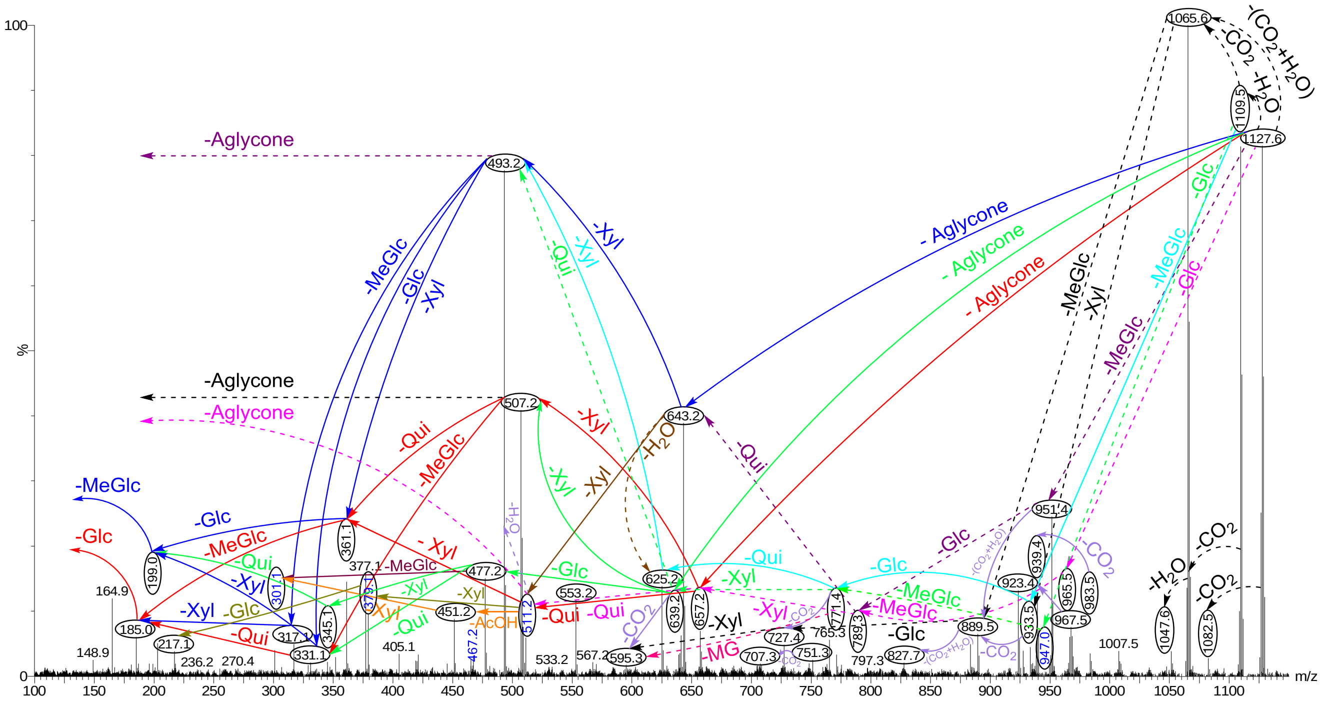

Abbreviations; MG = MeGlc, G = Glc, Q = Qui, X = Xyl.

(**b**)

Figure S3. Ion fragmentation pathways of the precursor ion at *m/z* 1227.4, and the proposed structures for the isomers.

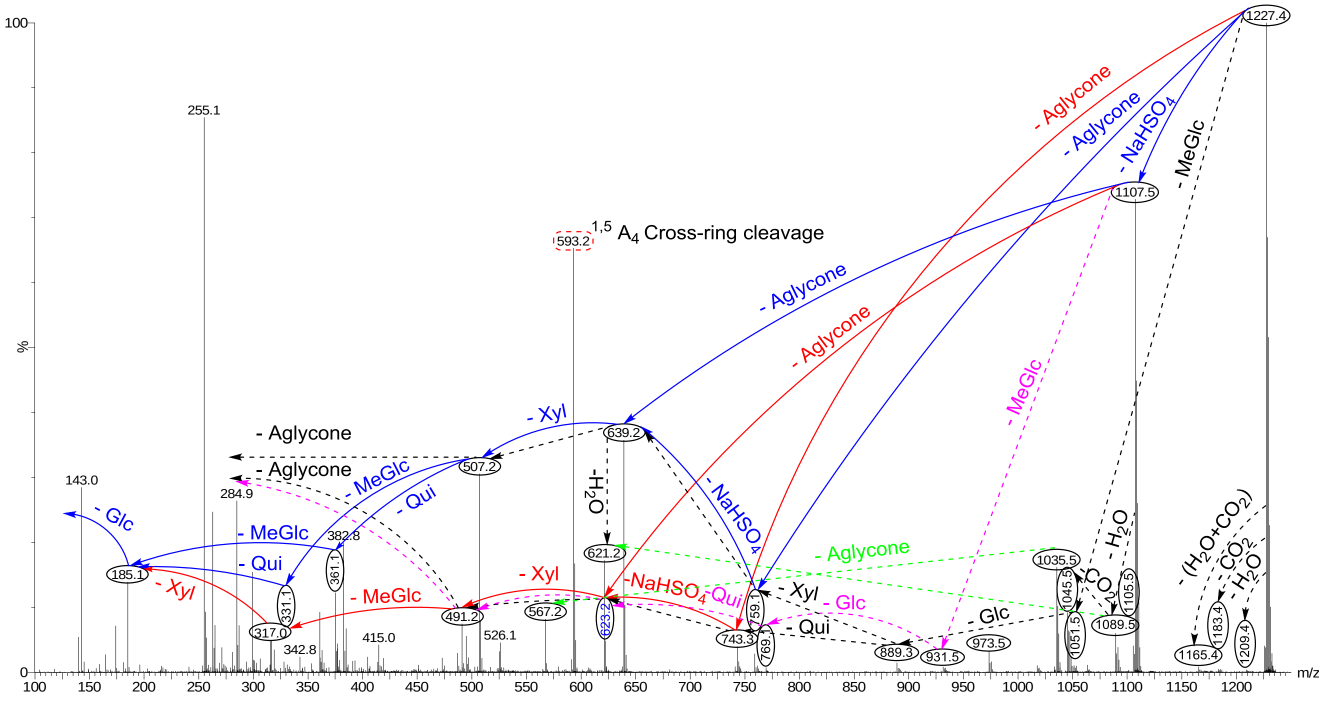

Figure S4. Ion fragmentation patterns of the precursor ion at *m/z* 1259.5 (**a**) ESI-MS/MS; (**b**) MALDI-MS/MS.

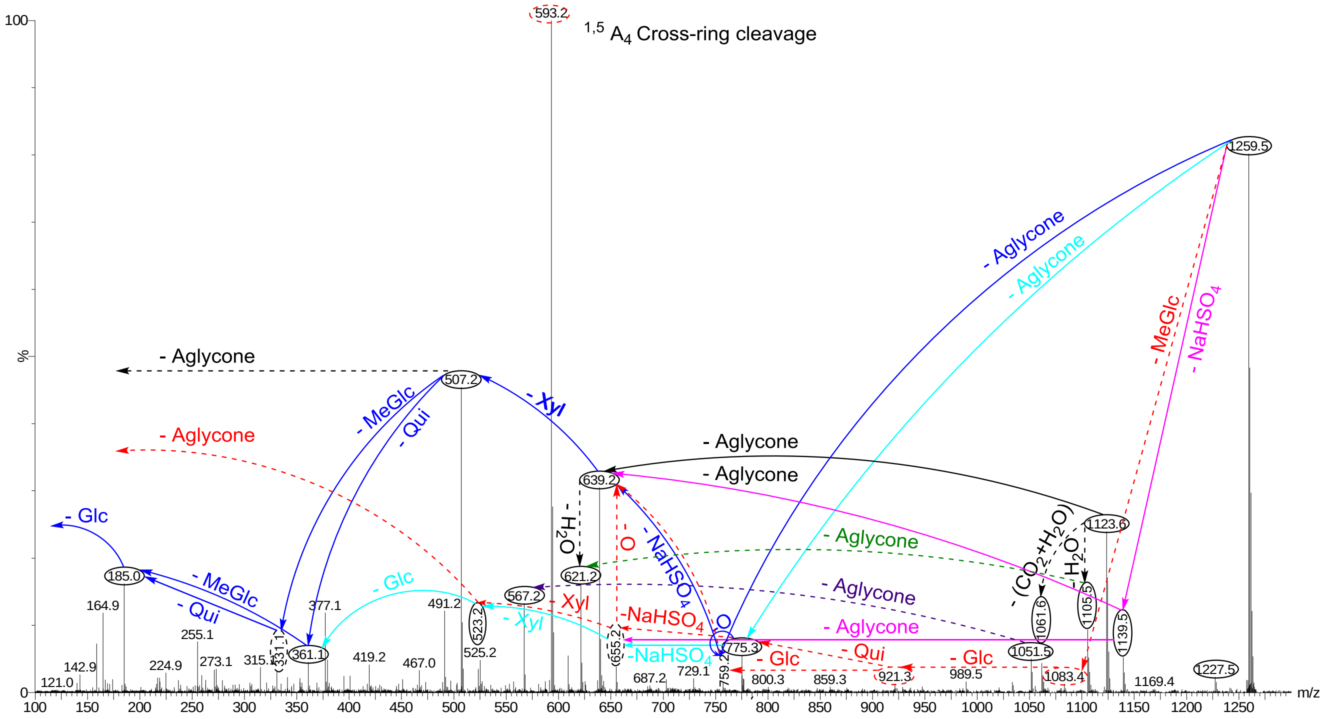

(**a**)

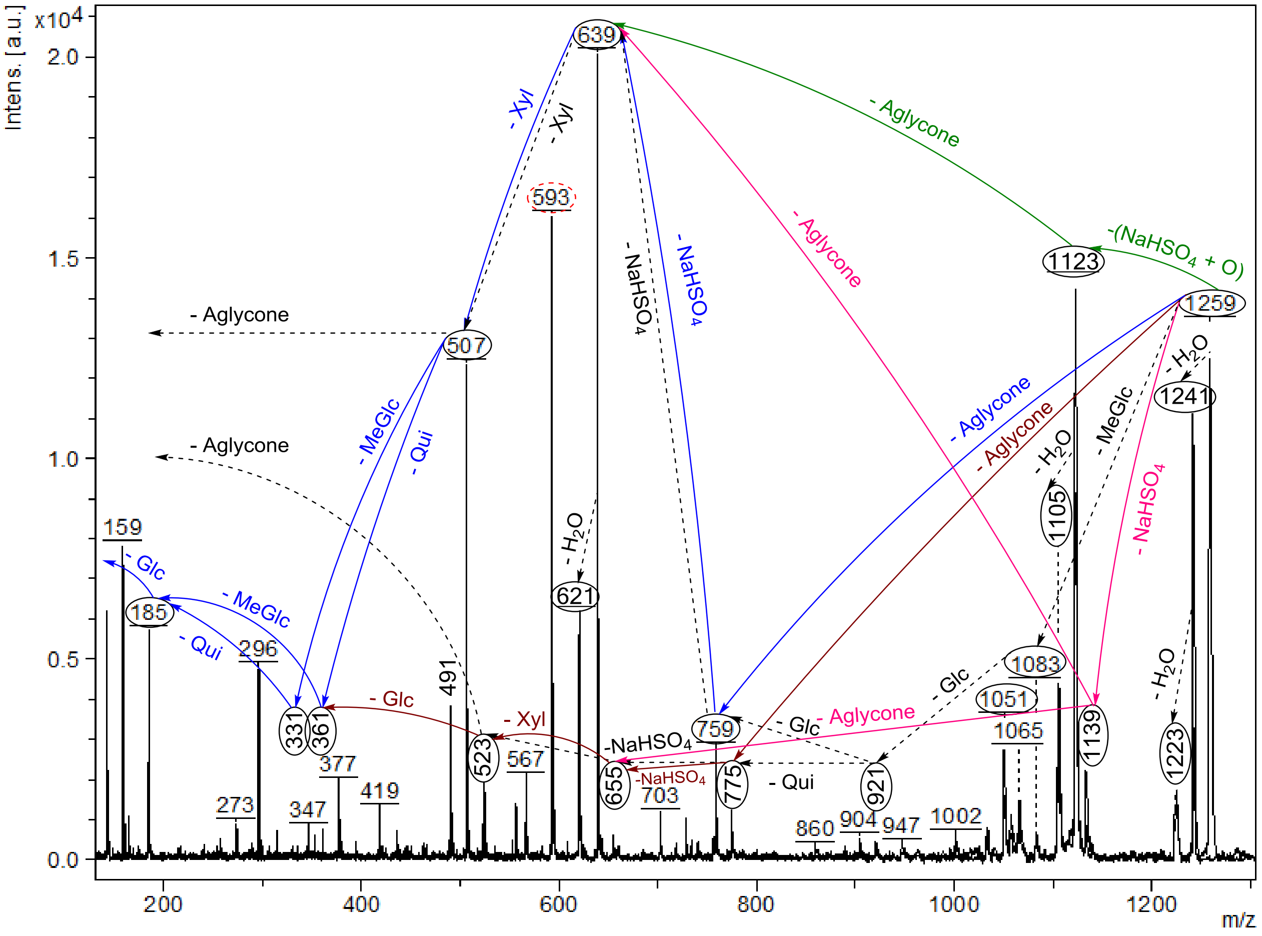

(**b**)

Figure S5. Proposed ion fragmentation and structures for the Holothurin A, corresponding to the precursor ion at *m/z* 1243.5, ESI-MS/MS (**a**); and MALDI-MS/MS (**b**).

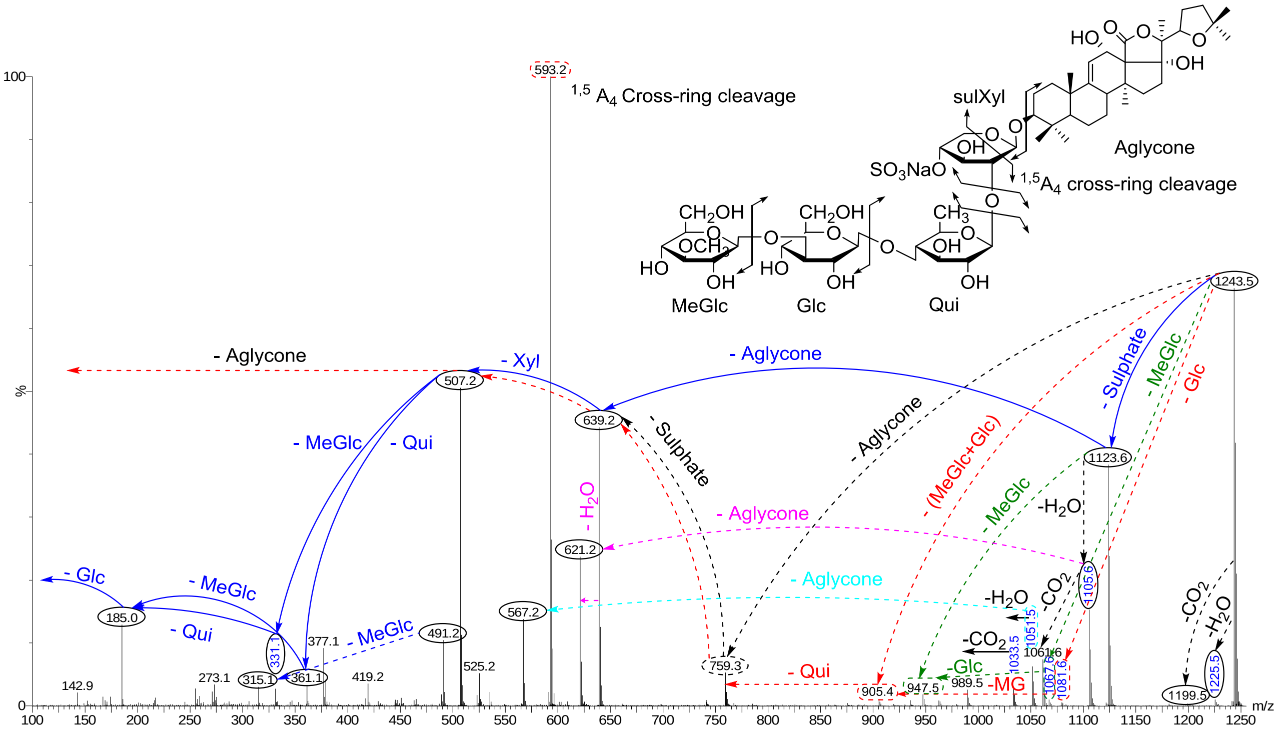

(a)

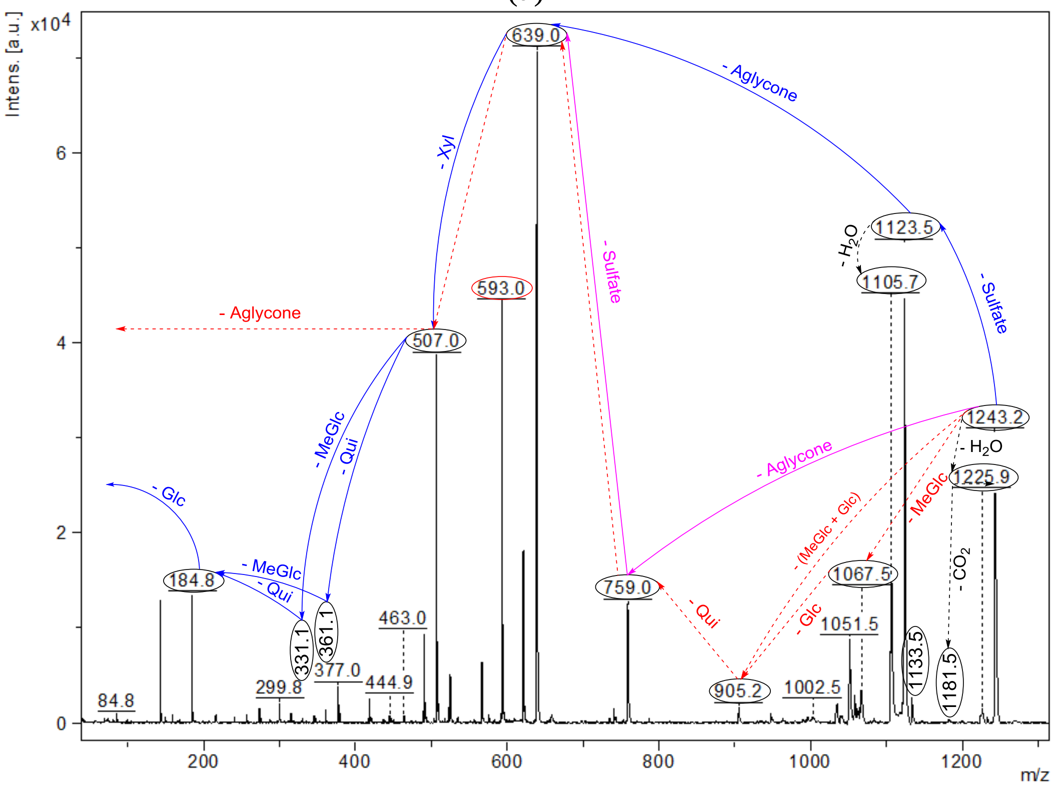

(b)

© 2014 by the authors; licensee MDPI, Basel, Switzerland. This article is an open access article distributed under the terms and conditions of the Creative Commons Attribution license (http://creativecommons.org/licenses/by/3.0/).
